# Genetic Predisposition to Multiple Myeloma at 5q15 Is Mediated by an *ELL2* Enhancer Polymorphism

**DOI:** 10.1016/j.celrep.2017.08.062

**Published:** 2017-09-12

**Authors:** Ni Li, David C. Johnson, Niels Weinhold, Scott Kimber, Sara E. Dobbins, Jonathan S. Mitchell, Ben Kinnersley, Amit Sud, Philip J. Law, Giulia Orlando, Matthew Scales, Christopher P. Wardell, Asta Försti, Phuc H. Hoang, Molly Went, Amy Holroyd, Fadi Hariri, Tomi Pastinen, Tobias Meissner, Hartmut Goldschmidt, Kari Hemminki, Gareth J. Morgan, Martin Kaiser, Richard S. Houlston

**Affiliations:** 1 Division of Genetics and Epidemiology, The Institute of Cancer Research, Surrey SM2 5NG, UK; 2 Division of Molecular Pathology, The Institute of Cancer Research, Surrey SM2 5NG, UK; 3 Myeloma Institute for Research and Therapy, University of Arkansas for Medical Sciences, Little Rock, AK 72205, USA; 4 Department of Internal Medicine V, University of Heidelberg, 69117 Heidelberg, Germany; 5 McGill University and Genome Quebec Innovation Centre, Department of Human Genetics, McGill University, Montreal, Quebec, QC H3A 0G1, Canada; 6 Department of Molecular and Experimental Medicine, Avera Cancer Institute, La Jolla, CA 92037, USA; 7 National Centre of Tumor Diseases, 69120 Heidelberg, Germany; 8 German Cancer Research Center, 69120 Heidelberg, Germany; 9 Center for Primary Health Care Research, Lund University, 205 02 Malmo, Sweden

**Keywords:** cancer genetics, genome-wide association studies, multiple myeloma, single nucleotide polymorphisms

## Abstract

Multiple myeloma (MM) is a malignancy of plasma cells. Genome-wide association studies have shown that variation at 5q15 influences MM risk. Here, we have sought to decipher the causal variant at 5q15 and the mechanism by which it influences tumorigenesis. We show that rs6877329 G > C resides in a predicted enhancer element that physically interacts with the transcription start site of *ELL2*. The rs6877329-C risk allele is associated with reduced enhancer activity and lowered *ELL2* expression. Since *ELL2* is critical to the B cell differentiation process, reduced *ELL2* expression is consistent with inherited genetic variation contributing to arrest of plasma cell development, facilitating MM clonal expansion. These data provide evidence for a biological mechanism underlying a hereditary risk of MM at 5q15.

## Highlights

• SNP rs6877329 underlies the association between 5q15 and multiple myeloma (MM)
• SNP rs6877329 forms a chromatin-looping interaction with the *ELL2* promoter
• rs6877329-C risk allele reduces enhancer activity in MM
• rs6877329-C confers lower *ELL2* expression in MM patients

## Graphical Abstract

**Figure:**
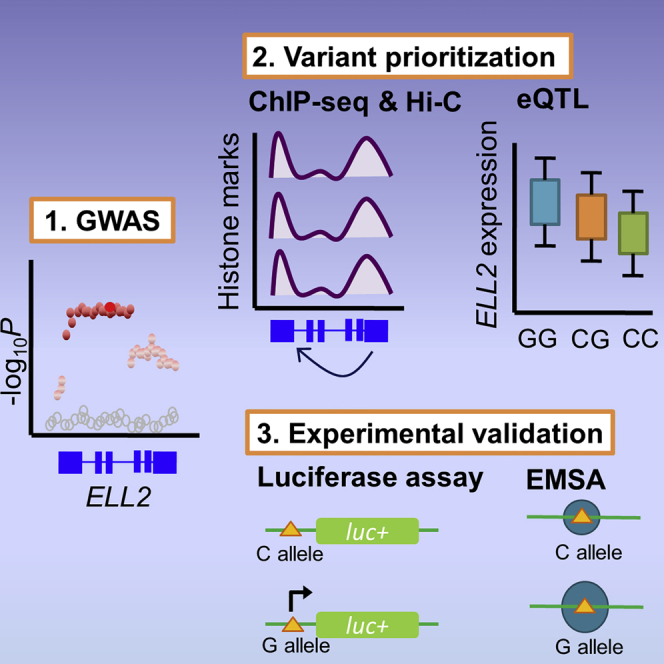

## Teaser

Li et al. find that rs6877329 underlies the 5q15 MM risk locus. Functional data demonstrate that rs6877329 resides within an enhancer that physically interacts with the *ELL2* promoter. The rs6877329-C risk allele reduces enhancer activity and is associated with reduced *ELL2* expression in MM patients.

## Introduction

Multiple myeloma (MM) is a malignancy of plasma cells primarily localized to the bone marrow (Kyle and Rajkumar, 2004, Kyle et al., 2007). The disease is genetically heterogeneous but can be broadly divided into hyperdiploid MM (HRDMM) and non-HRDMM subtypes (Gould et al., 1988, Sawyer et al., 1995, Smadja et al., 1998). Non-HRDMM is characterized by translocations of the immunoglobulin heavy chain (*IgH*) alleles at 14q32 with various recurrently observed genes, the significance of which is generally considered to be increased expression of the translocated partner gene. In contrast, HRDMM involves trisomies of odd-numbered chromosomes.

Although the etiological basis of MM is poorly understood, it has a significant genetic component as evidenced by a 2- to 4-fold increased risk in first-degree relatives of MM patients (Altieri et al., 2006, Landgren et al., 2009, Vachon et al., 2009). Our understanding of MM susceptibility has recently been transformed by genome-wide association studies (GWASs), which provide strong evidence that common genetic variation influences MM risk. So far, GWASs have identified 17 independent risk loci, with the signal annotating elongation factor for RNA polymerase II 2 (*ELL2*) at 5q15 being highly robust (Broderick et al., 2011, Chubb et al., 2015, Mitchell et al., 2016, Swaminathan et al., 2015). *ELL2* encodes a key component of the super-elongation complex (SEC) that drives secretory-specific immunoglobulin (Ig) mRNA production and transcriptional regulation in plasma cells (Park et al., 2014).

Here, we sought to identify the causal polymorphism(s) driving the 5q15 genetic association with MM susceptibility as a basis for understanding MM initiation. Our data are compatible with the rs6877329 variant as the functional basis of the 5q15 association, a genomic region, which through chromatin-looping interaction leads to the reduced expression of *ELL2*.

## Results

### Fine Mapping and Epigenomic Profiling of the 5q15 Locus

**Figure 1:**
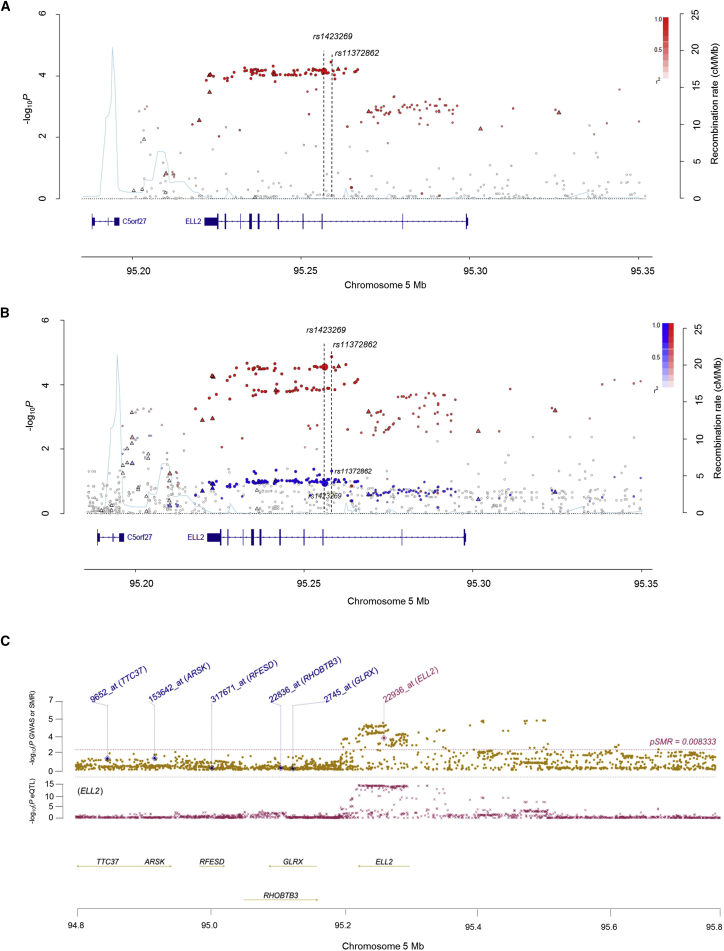
Regional Plots of Association Results of the 5q15 Locus (A and B) The region of association maps to a ∼40 kb haplotype block within *ELL2*. Genotyped (triangles) and imputed (dots) SNPs are shown based on their chromosomal position (NCBI build 37 human genome) on the x axis and −log_10_ p value on the y axis from (A) GWAS meta-analysis in UK MRC MyIX and MyXI trials and German GMMG trials (3,790 case subjects and 7,304 control subjects), and (B) HRDMM (red) and non-HRDMM (blue) case-control meta-analysis in the UK and German populations (1,363 HRDMM case subjects, 1,339 non-HRDMM case subjects, and 7,304 control subjects). Color intensity of each SNP reflects the extent of LD with the lead SNP, rs11372862 (white *r*^*2*^ = 0 to dark red/blue *r*^*2*^ = 1). Recombination rates, estimated using HapMap samples of European ancestry, are shown by a light blue line. The relative positions of *ELL2* and *C5orf27* mapping to 5q15 are shown. rs1423269 and rs11372862 are annotated in black dotted lines. (C) Summary data-based Mendelian randomization analysis at 5q15. Upper panel - brown dots represent p values for SNPs from the HRDMM case-control meta-analysis (1,363 cases and 7,304 controls), diamonds represent p values for probes from the SMR test; lower panel - crosses represent eQTL p values of SNPs from MM plasma cells from 183 MRC MyIX trial patients (GEO: GSE21349) and 658 Heidelberg GMMG patients (EMBL-EBI: E-MTAB-2299), with genes passing the SMR (i.e., *P*_*SMR*_ < 0.00833) and HEIDI (i.e. *P*_*HEIDI*_ > 0.05) tests highlighted in red. Probeset ID refers to Affymetrix U133 2.0 Plus Array custom chip definition file (CDF v.17) mapping to Entrez genes.

We analyzed previously published UK and German GWAS datasets totaling 3,790 case subjects and 7,304 control subjects (Mitchell et al., 2016), genotyped on Illumina Human OmniExpress-12 v1.0, Illumina HumanOmni1-Quadv1, and Hap1.2M-Duo Custom arrays. To inform fine-mapping of the 5q15 risk locus, we imputed untyped genotypes in both GWASs using the UK10K (Huang et al., 2015) and 1000 Genomes Project (The 1000 Genomes Project Consortium, 2010) as a reference. Overall, the strongest association across all forms of MM in a meta-analysis of the two GWAS datasets was provided by rs11372862 (OR = 1.16, p = 3.75 × 10^−5^, *P*_*het*_ = 0.85, Figure 1A), which is highly correlated with rs1423269 (*r*^*2*^ = 0.97, *D’* = 0.97), the previously reported sentinel SNP for the 5q15 risk locus. Conditional analysis provided no evidence for additional independently associated SNPs at 5q15. By referencing germline whole-genome sequencing (WGS) data on 640 MM patients analyzed as part of the CoMMpass Study (Craig et al., 2013), we were able to confirm that the imputation captured >90% of sequence variation (minor allele frequency [MAF] > 0.05) within the linkage disequilibrium (LD) region encompassing rs11372862 (i.e., pairwise *r*^*2*^ ≥ 0.1) (Table S1). By analyzing the germline exomes of 513 MM case subjects from the UK Medical Research Council (MRC) MyIX and MyXI clinical trials and 1,569 UK control subjects from the UK 1958 Birth Cohort (Scales et al., 2017), we excluded the possibility that the 5q15 association signal is a consequence of LD with a rare disease-causing coding variant (Table S2).

Since there is previous evidence of subtype specificity for MM association (Weinhold et al., 2013), with the 11q13.3 association for MM being highly specific for t(11;14) MM, we examined whether the 5q15 association might also show evidence of subtype specificity. Stratifying MM by subtype revealed that risk at 5q15 was primarily associated with HRDMM (OR = 1.26, p = 1.37 × 10^−5^, *P*_het_ = 0.52) (Figure 1B; Table S3A). Case-only analysis also provided supportive evidence that the rs11372862 association was mainly driven by HRDMM (p = 0.04; Table S3B).

To gain further insight into the association, we performed an expression quantitative trait locus (eQTL) analysis using mRNA expression data on CD138-purified plasma cells from 841 MM case subjects from the UK MRC MyIX trial and German-Speaking Multiple Myeloma Multicenter Study Group (GMMG) trials. Specifically, we used Summary data-based Mendelian randomization (SMR) analysis to test for pleiotropy between GWAS signal and *cis*-eQTL for genes within 1 Mb of the lead SNP rs11372862 to identify a causal relationship (Zhu et al., 2016). eQTL analysis provided evidence for differential *ELL2* expression as being the basis of the 5q15 association (Figure 1C; Figure S1). Multiple SNPs (n = 90, including rs1423269, rs11372862, rs3777184, and rs6877329) mapping within *ELL2* in strong LD essentially defined a single haplotype defining MM risk and eQTL (Figure 1C).

**Figure 2:**
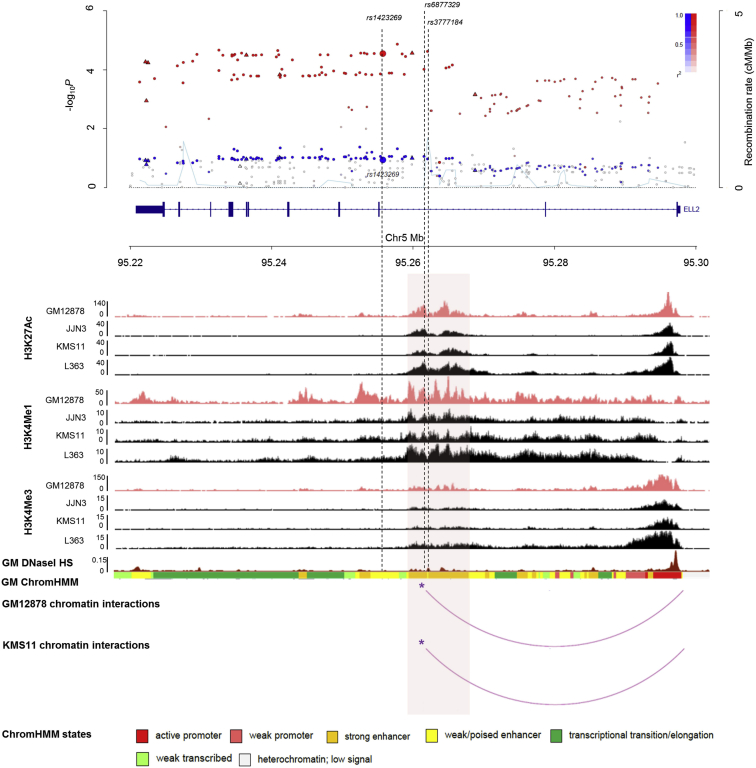
Epigenetic Landscape at the 5q15 Locus HRDMM (red) and non-HRDMM (blue) case-control meta-analysis as shown in Figure 1B. ChIP-seq from GM12878 (pink peaks), JJN3, KMS11, and L363 (black peaks) are shown, annotated with the ChIP’d histone modification marks H3K4Me1, H3K4Me3, and H3K27Ac. DNaseI HS and ChromHMM data for GM12878 were assessed from ENCODE. A ∼8kb active enhancer (chr5:95,259,093-95,267,656) within *ELL2* is shaded, predicted by ChromHMM, DNaseI HS, as well as H3K4Me1, H3K4Me3, and H3K27Ac peaks. rs3777185, rs6877329, rs3777184, rs889302, rs2015159, and rs4563648 are localized within the enhancer with *r*^*2*^ ≥ 0.8 with rs11372862 (Table S3). Asterisk (^∗^) marks the enhancer region interacting with the *ELL2* promoter in both GM12878 and KMS11 (chr5:95,260,175-95,264,576) encompassing rs6877329 and rs3777184. The positions of rs1423269, rs6877329, and rs3777184 are marked with black dotted lines.

The eQTL data suggest the 5q15 association with MM is likely to be mediated by the regulation of *ELL2* expression. To prioritize candidate risk variants, we examined the SNPs in LD (*r*^*2*^ ≥ 0.8) with rs11372862 within regulatory elements defined by B cell-specific DNase I hypersensitivity (DNaseI HS) and promoter/enhancer-associated histone marks (Figure 2). Six SNPs each correlated with rs11372862 localize within an 8 kb active enhancer region (Table S4), supported by ChromHMM, open chromatin analysis (DNaseI HS), as well as H3K4Me1, H3K4Me3, and H3K27Ac peaks in GM12878 (ENCODE Project Consortium, 2012), the MM cell line KMS11, and the plasma cell leukemia cell lines (PCL) JJN3 and L363.

Physical interactions between regulatory elements and promoters play a major role in regulating gene expression (Mifsud et al., 2015, Rao et al., 2014). Following our observation that six correlated SNPs localize within an enhancer element, we interrogated whether this genomic region physically interacts with the *ELL2* promoter in both KMS11 and GM12878 using promoter capture Hi-C (CHi-C) data. Within the active enhancer, rs6877329 and rs3777184 fall within an overlapping genomic fragment in both cell lines, forming a chromatin-looping interaction with the *ELL2* promoter (Figure 2; Table S4).

### Effect of rs6877329 and rs3777184 Genotypes on Enhancer Activity

**Figure 3:**
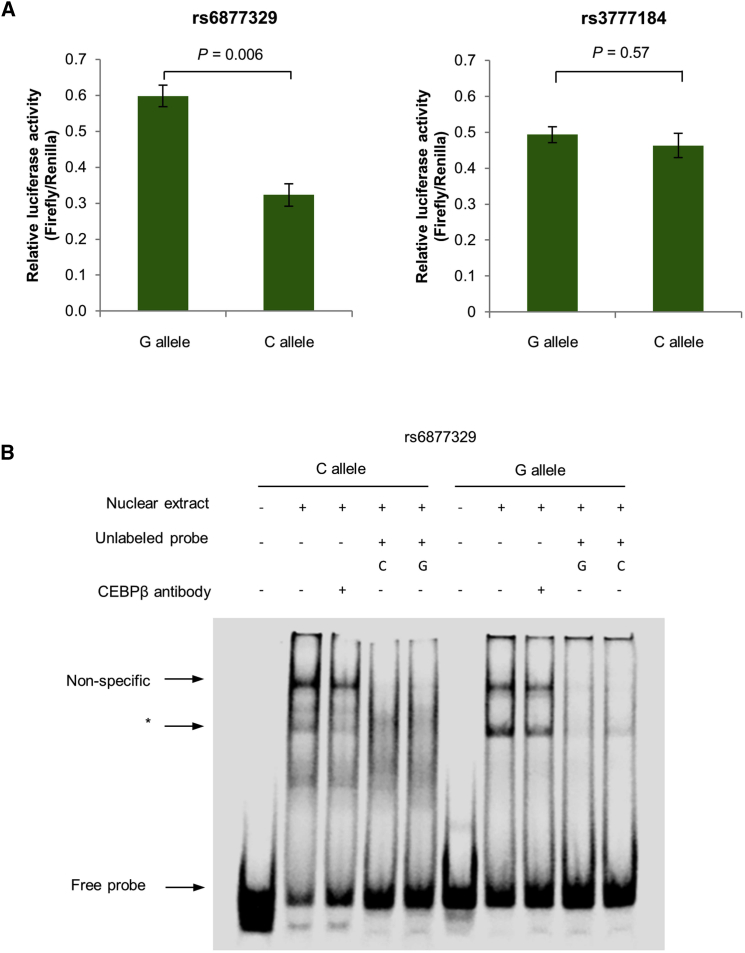
rs6877329 Is Associated with Reduced Enhancer Activity and Differential Nuclear Protein Binding (A) rs6877329-C risk allele shows decreased expression over the protective G-allele. Allele-specific constructs containing a putative regulatory sequence flanking rs6877329 and rs3777184 were cloned into the pGL3 *luc+* SV40-promoter vector and transfected into KMS11. The ratio of luminescence from the experimental pGL3-constructs to the Renilla internal control, pRL-SV40, was normalized to the empty pGL3 *luc+* SV40-promoter vector. Data shown are mean ± SEM from three independent experiments performed in triplicate. Differences in gene expression were assessed by the two-tailed t test. (B) Electrophoretic mobility shift assay in GM11992 showing differential nuclear protein binding to alleles of rs6877329. Increased protein binding observed for the non-risk G-allele. Asterisk (^∗^) indicates specific protein shift band.

To measure the effect of rs6877329 and rs3777184 alleles on enhancer activity, we performed luciferase reporter assays in KMS11. Transfection with constructs containing the rs6877329-C risk allele displayed significantly lower normalized luminescence compared to non-risk G-allele construct (two-tailed t test p = 0.006, Figure 3A). rs3777184 genotype did not influence enhancer activity (two-tailed t test p = 0.57, Figure 3A). These data are thus consistent with a model of MM risk in which variation at rs6877329 is associated with decreased expression of *ELL2* (Table S5A). We next assayed protein-DNA interactions for rs6877329-C and rs6877329-G alleles using an electrophoretic mobility shift assay (EMSA). The G-allele formed stronger protein-DNA complexes compared with the C-allele, consistent with the region having differential transcription factor (TF) binding (Figure 3B). From ENCODE chromatin immunoprecipitation sequencing (ChIP-seq) data on GM12878, the CCAAT/enhancer-binding protein beta (CEBPB) is the only TF overlapping with rs6877329, albeit marginally. We did not, however, demonstrate an EMSA super-shift with CEBPB antibody with the G- and C-alleles (Figure 3B).

### rs6877329 Risk Allele Is Not Preferentially Amplified in Hyperdiploid Myeloma

Trisomy of chromosome 5 typifies HRDMM. To investigate a possible relationship between heritable risk associated with rs6877329 and somatic mutation, we sought to determine whether the risk C-allele, associated with reduced *ELL2* expression, is preferentially amplified in HRDMM. We analyzed whole-exome sequencing (WES) data on two independent series of HRDMM tumors that were trisomic for chromosome 5 and heterozygous for rs6877329, using the highly correlated synonymous SNP rs3777204 and the missense SNP rs3815768 as proxies (rs3777204 *r*^*2*^ = 0.99, *D’* = 1.00; rs3815768 *r*^*2*^ = 0.95, *D’* = 0.99). rs3815768 is predicted to be benign and tolerated by PolyPhen-2 and SIFT, respectively. After correcting for the germline ratio of reference to alternate alleles in both series, we found no evidence of preferential duplication of the risk C-allele (Table S6).

### *ELL2* Expression Correlates with Unfolded Protein Response and SEC Components

**Figure 4:**
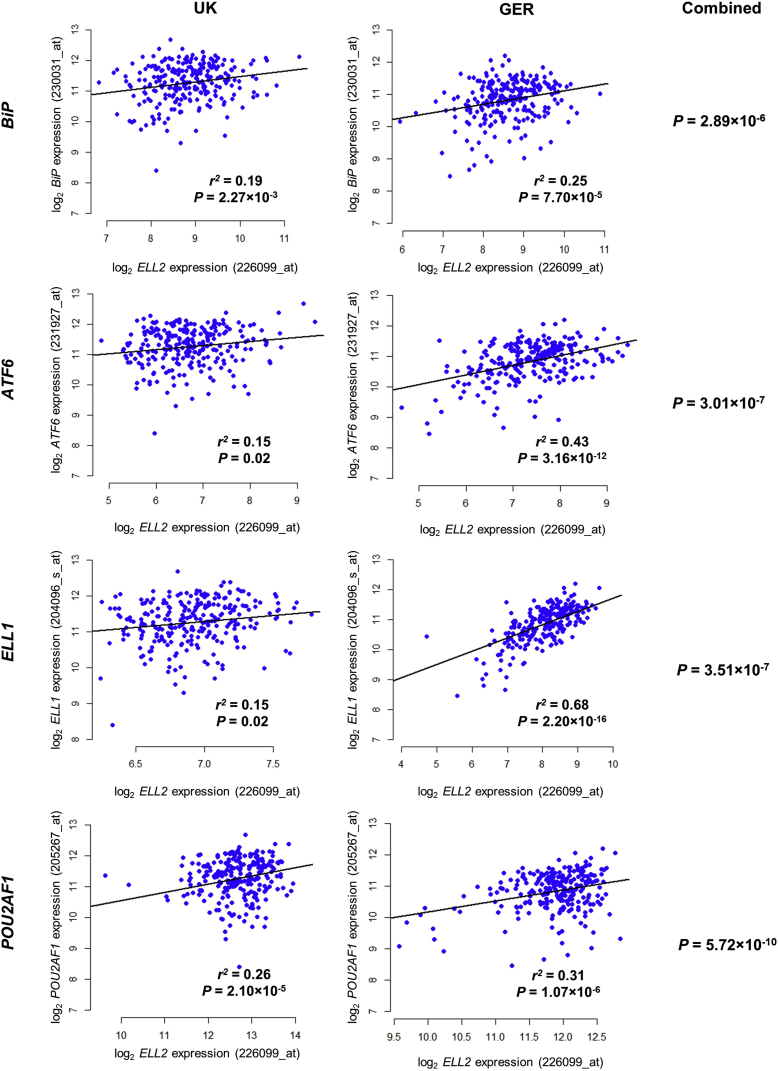
Gene Expression Correlations with *ELL2* in Multiple Myeloma Expression correlation between *ELL2* and *BiP,* and *ATF6, ELL1,* and *POU2AF1* was examined in 259 UK MM patients (GEO: GSE21349) and 246 German (GER) MM patients (EMBL-EBI: E-MTAB-372). Lines show linear regression fits. Expression correlation was assessed by Pearson’s product-moment correlation test. p values were combined from independent datasets using Fisher’s Method.

We sought to establish a possible functional consequence of reduced *ELL2* expression on MM oncogenesis. A recent study of conditional knockout (cKO) *ELL2* mice identified 10 genes where loss of *ELL2* expression resulted in at least a 2-fold difference in expression (Park et al., 2014). We assessed mRNA expression correlation between *ELL2* and these 10 genes in the tumors from 505 MM patients (GEO: GSE21349 from the UK MRC MyIX trial; EMBL-EBI: E-MTAB-372 from the German GMMG trial). Although there was no evidence for a *trans*-eQTL in *BiP* (binding immunoglobulin protein), *ATF6* (activating TF 6), *ELL1* (elongation factor for RNA polymerase II, 1), and *POU2AF1* (POU class 2 associating factor 1) (Table S5B), these genes consistently correlated with *ELL2* expression in MM (Figure 4).

### Impact of rs6877329 Genotype and Gene Expression on Patient Prognosis

To examine the relationship between rs6877329 genotype and *ELL2* expression on patient outcome, defined by overall survival (OS) and progression-free survival (PFS), we made use of data from the UK MRC MyIX and MyXI trials and the German GMMG trial, totaling 505 MM patients with expression data and 2,553 patients with genotype data. Meta-analysis of these data provided no evidence for an association between either rs6877329 genotype or the level of *ELL2* expression with either OS or PFS (p > 0.4; Tables S7A and S7B). We also found no evidence linking *BiP*, *ATF6*, *ELL1,* and *POU2AF1* expression to patient outcome (p > 0.2; Tables S7C–S7F).

## Discussion

We acknowledge that the large LD block at 5q15 presents limitations in prioritizing the functional variant underscoring the association. However, collectively our data demonstrate a plausible mechanism underlying MM risk being mediated through rs6877329, compatible with a differential effect on TF binding. Moreover, data are compatible with the rs6877329-C allele conferring increased risk through reduced expression of *ELL2*. Epigenetic and chromosome conformation capture data are consistent with rs6877329 localizing within a chromatin contact domain and overlapping a B cell enhancer. This interval forms a “loop domain,” bringing it into physical contact close to the transcription start site of *ELL2*, separated by a linear distance of around 35 kb.

*ELL2* plays a key role in the differentiation of mature B cells into plasma cells (Park et al., 2014). *ELL2* is induced 6-fold in plasma cells and drives secretory-specific *IgH* mRNA production via enhanced exon skipping and polyadenylation (Martincic et al., 2009). B cell lineage *ELL2* cKO mice show reduced numbers of plasma cells and a paucity of secreted Ig (Park et al., 2014). We have shown that individuals carrying the rs6877329-C risk allele have reduced *ELL2* transcript levels. The observation that the MM risk locus at 5q15 is associated with reduced levels of IgA and IgG in healthy individuals is thus consistent with a hypomorphic effect associated with reduced *ELL2* expression (Swaminathan et al., 2015). These data do not lend themselves to an obvious basis for allele-specific reduction in *ELL2* being associated with increased MM risk.

We observed a strong relationship between *ELL2* with *BiP* and *ATF6* expression in MM. *BiP* is a regulator of the UPR pathway during endoplasmic reticulum (ER) stress—a pathway heavily relied on by MM plasma cells for survival due to its active production and secretion of immunoglobulins (Vincenz et al., 2013). *BiP* has been associated with the activation of UPR inducers, such as *ATF6*, *PERK* (PKR-like ER kinase), and *IRE1* (inositol-requiring enzyme 1) (Bertolotti et al., 2000). Furthermore, the UPR pathway regulates the equilibrium between proliferation and cell death during ER stress, and the formation of autophagosome can be inhibited by *BiP* knockdown (Li et al., 2008). Collectively, these data are compatible with *ELL2* having a role in the UPR and autophagy regulation in MM through its interaction with *BiP* and *ATF6*. Importantly, due to the pre-existing ER stress in MM plasma cells, such cells are particularly prone to drug-induced ER stress by, for example, the proteasome inhibitor bortezomib (Obeng et al., 2006). *ELL2* is also associated with *ELL1* and *POU2AF1* expression in MM patients, the latter being a transcriptional coactivator of *OCT2* (octamer-binding protein 2), expression of which is required for B cell differentiation (Hodson et al., 2016). Reduced *ELL2* expression associated with 5q15 thus potentially suggests an impairment of SEC function and hindrance in plasma cell development.

We did not observe an association between rs6877329 genotype or *ELL2* expression level on patient survival in MM. While our analysis had 80% power to demonstrate a 10% difference in patient outcome at p = 0.05, we acknowledge that to detect a smaller impact would require much larger patient cohorts. Accepting this caveat, our findings are consistent with differential expression of *ELL2* being important in the early phase of MM tumor development rather than disease progression per se.

To directly inform of a possible relationship between the rs6877329-C risk allele and somatic mutation, we exploited the fact that the 5q15 association was primarily shown for HRDMM. The effects of chromosome 5 gain are likely driven by a selective advantage conferred by increased dosage of additional unknown factors unrelated to the primary function of *ELL2*. Evidence that HRDMM heterozygotes preferentially duplicate the chromosome 5 homolog with the rs6877329-C risk allele would have potentially suggested increased dosage of chromosome 5 gene expression relative to *ELL2*. Irrespective of a failure to demonstrate such a relationship and although speculative, it is possible that decreased *ELL2* expression would increase the probability of arrest of normal plasma cell development, facilitating MM clonal expansion. Since the association between rs6877329-C risk allele and *ELL2* expression is not exclusive to HRDMM, these data suggest that loss of *ELL2* activity is relevant in the context of both HRDMM and non-HRDMM primary initiating events.

In conclusion, we have shown reduced *ELL2* expression in MM patients carrying the rs6877329-C risk allele, thus providing a mechanistic basis for the 5q15 risk association for MM. Further functional studies, however, are required to fully decipher the biological basis of differential *ELL2* expression on MM oncogenesis.

## Experimental Procedures

### Ethics

Collection of patient samples and associated clinico-pathological information was undertaken with written informed consent and relevant ethical review board approval at respective study centers in accordance with the tenets of the Declaration of Helsinki, specifically, the MRC Leukemia Data Monitoring and Ethics Committee (MREC 02/8/95, ISRCTN68454111, MREC 17/09/09, and ISRCTN49407852) and the University of Heidelberg Ethical Commission (229/2003, S-337/2009, AFmu-119/2010).

### GWAS Data

The UK-GWAS and German-GWAS of MM have been previously reported (Mitchell et al., 2016). The diagnosis of MM (ICD-10 C90.0) was established in accordance with World Health Organization guidelines. All samples from patients for genotyping were obtained before treatment or at presentation. The UK-GWAS comprised 2,329 MM case subjects (1,060 male; mean age at diagnosis: 64.0 years), including 702 with HRDMM, recruited through the UK MRC MyIX and MyXI trials. Control subjects were provided by the Wellcome Trust Case Control Consortium 2 with 2,698 individuals in the 1958 British Birth Cohort and 2,501 individuals from the UK Blood Service. The German-GWAS comprised 1,512 MM case subjects (867 male; mean age at diagnosis: 59 years), including 661 with HRDMM. Case subjects were recruited by the GMMG trial. Control subjects comprised 2,107 healthy individuals from the Heinz Nixdorf Recall study. To recover untyped genotypes, we performed imputation using IMPUTE2 v2.3 with a combined UK10K and 1000 Genomes Project (phase 1 integrated release 3, March 2012) panel for reference (The 1000 Genomes Project Consortium, 2010, Huang et al., 2015). Poorly imputed SNPs (INFO score < 0.80) were excluded. Frequentist association testing between SNP genotype and MM was performed using logistic regression under an additive genetic model in SNPTESTv2.5 (Marchini et al., 2007). Meta-analysis was undertaken under a fixed-effects model using inverse variance weighting in METAv1.7 (Marchini et al., 2007). To look for independent effects, we performed conditional analysis with SNPTESTv2.5 with genotypes from UK and German GWAS individuals conditioning on rs11376892. Logistic regression in case-only and case-control analyses was used to assess tumor subtype.

### Expression Quantitative Trait Loci Analysis

eQTL analyses were performed for genes and SNPs within 1 MB of rs11372862 for CD138-purified plasma cells from 183 UK MyIX trial patients and 658 German GMMG patients. Briefly, German and UK data were pre-processed separately, followed by analysis using a Bayesian approach to probabilistic estimation of expression residuals to infer broad variance components, accounting for hidden determinants influencing global expression. The association between genotype of SNPs and expression of genes within 500 kb either side of rs11372862 was evaluated based on the significance of linear regression coefficients. We pooled data from the two studies under a fixed-effects model. Subtype-specific eQTL analyses were performed for *ELL2, BiP, ATF6,* and *POU2AF1* expression and rs11372862, rs1423269, and rs6877329 for 170 UK MyIX trial and 602 German GMMG patients with subtype and expression data. We carried out SMR analysis using previously established methods (Zhu et al., 2016), with a threshold for the SMR test set at *P_SMR_* < 0.00833 corresponding to Bonferroni correction.

### ENCODE and Chromatin State Dynamics

To explore the epigenetic profile of association signals at 5q15, we used DNaseI HS, TF ChIP-seq data, histone modifications (H3K4Me1, H3K4Me3, and H3K27Ac), and ChromHMM in GM12878 from the ENCODE project (ENCODE Project Consortium, 2012). ChIP-seq on H3K4Me1, H3K4Me3, and H3K27Ac were carried out in KMS11, L363, and JJN3 cell lines.

### In Situ Promoter Capture Hi-C

In situ promoter capture Hi-C libraries were prepared for KMS11 as previously described (Rao et al., 2014). The interaction within *ELL2* from its promoter with the highest score was plotted. Promoter capture Hi-C on GM12878 was obtained from EMBL-EBI: E-MTAB-2323 (Mifsud et al., 2015).

### Plasmid Construction and Luciferase Assays

Regulatory region with rs6877329 and rs3777184 risk/non-risk alleles was cloned into pGL3 *luc+* SV40-promoter vector (Promega, Madison, WI, USA). Reporter constructs were introduced into KMS11. Relative luciferase activity was calculated as the ratio of luminescence from the experimental reporter to the internal control plasmid (pRL-SV40). We calculated statistical significance by using the two-tailed t test over three biological replicates.

### Electrophoretic Mobility Shift Assay

Nuclear protein from GM11992 cells was incubated with infrared dye DY-682-labeled double-stranded EMSA probes flanking rs6877329 (Eurofins Genomics, Germany) (Supplemental Experimental Procedures). Competition assays were performed by adding 100-fold molar excess of unlabeled probes. Super-shifts were performed by adding 2 μg CEBPB antibody (sc-376591; Santa Cruz Biotechnology, Texas, USA). DNA-protein complexes were resolved by electrophoresis on a 6% DNA retardation gel (Life Technologies, Carlsbad, CA, USA) in 1 × Tris-borate-EDTA (TBE).

### Gene Expression Analyses

We assessed the relationship between *ELL2* and *BiP*, *ATF6*, *POU2AF1*, and *ELL1* gene expression (log_2_-transformed) in 259 UK MyIX clinical trial patients and 246 German GMMG clinical trial patients using Pearson’s product-moment correlation test. p values from the two patient datasets were combined by Fisher’s Method. We conducted statistical tests using the R software version 3.1.3.

### Relationship between SNP Genotype and Somatic Copy Number

To investigate whether rs6877329 was preferentially amplified in heterozygous individuals with chromosome 5 trisomy, we analyzed WES data on 463 UK MyXI trial cases (Walker et al., 2015) in conjunction with WGS and WES data on 664 cases produced by the MM CoMMpass Study (Craig et al., 2013). 57 and 50 hyperdiploid samples heterozygous for the risk variant with chromosome 5 trisomy were identified in the MyXI and CoMMpass datasets, respectively. Given the counts of risk and non-risk reads sampled at two proxy coding SNPs (rs3777204 and rs3815768, *r*^*2*^ > 0.95), each sample was assigned to its most probable state (amplification of the risk or non-risk allele) assuming a binomial distribution of counts and adjusting for reference mapping bias using germline read counts.

### Association between rs6877329 Genotypes, *ELL2* Expression, and Patient Outcome

The relationship between *ELL2, BiP, ATF6, ELL1,* and *POU2AF1* expression and patient outcome (OS and PFS) was assessed with 259 UK MyIX clinical trial patients and 246 German GMMG clinical trial individuals grouped by their *ELL2* expression (upper and lower quartiles). Analysis was performed using the log-rank test to estimate expression-associated hazard ratio and the 95% confidence interval. Statistical tests were conducted using the R software version 3.1.3. We assessed the relationship between rs6877329 genotype and patient outcome using GWAS data on (1) 1,165 patients in the UK GWAS from the MyIX trial, (2) 877 MM patients in the UK GWAS from the MyXI trial, and (3) 511 of the patients in the German GWAS from the GMMG clinical trial. Cox regression analysis was used to derive genotype-specific hazard ratio and associated 95% confidence intervals.

## Author Contributions

N.L. and R.S.H. drafted the manuscript; M.K. contributed. N.L. and R.S.H. designed the study. N.L., S.K., G.O., and A.H. performed laboratory work. F.H. performed ChIP-seq experiments. H.G., G.J.M., K.H., and M.K. performed sample ascertainment and provided data. N.L., D.C.J., N.W., S.E.D., J.S.M., A.S., P.J.L., B.K., M.S., C.P.W., A.F., P.H.H., M.W., and T.M. performed bioinformatics and statistical analyses. All authors contributed to the final manuscript.

## Data and Software Availability

The accession number for the ChIP-seq data reported in this paper is EGA: S00001002414. The accession number for the Hi-C data reported in this paper is EGA: S00001002614.

## Supplemental Information

Document S1. Supplemental Experimental Procedures, Figure S1, and Tables S1–S7

Document S2. Article plus Supplemental Information
