# Supplementary material for: Genetic Predisposition to Multiple Myeloma at 5q15 Is Mediated by an ELL2 Enhancer Polymorphism
Source: Cell Rep. 2017 Sep 12;20(11):2556–64. doi: 10.1016/j.celrep.2017.08.062 (PMC5608969; doi:10.1016/j.celrep.2017.08.062)
Supplement: Document S1. Supplemental Experimental Procedures, Figure S1, and Tables S1–S7 [file mmc1.pdf]

Cell Reports, Volume *20*

Supplemental Information

Genetic Predisposition to Multiple Myeloma at 5q15 Is Mediated by an *ELL2* Enhancer Polymorphism

Ni Li, David C. Johnson, Niels Weinhold, Scott Kimber, Sara E. Dobbins, Jonathan S. Mitchell, Ben Kinnersley, Amit Sud, Philip J. Law, Giulia Orlando, Matthew Scales, Christopher P. Wardell, Asta Försti, Phuc H. Hoang, Molly Went, Amy Holroyd, Fadi Hariri, Tomi Pastinen, Tobias Meissner, Hartmut Goldschmidt, Kari Hemminki, Gareth J. Morgan, Martin Kaiser, and Richard S. Houlston

Supplemental Information

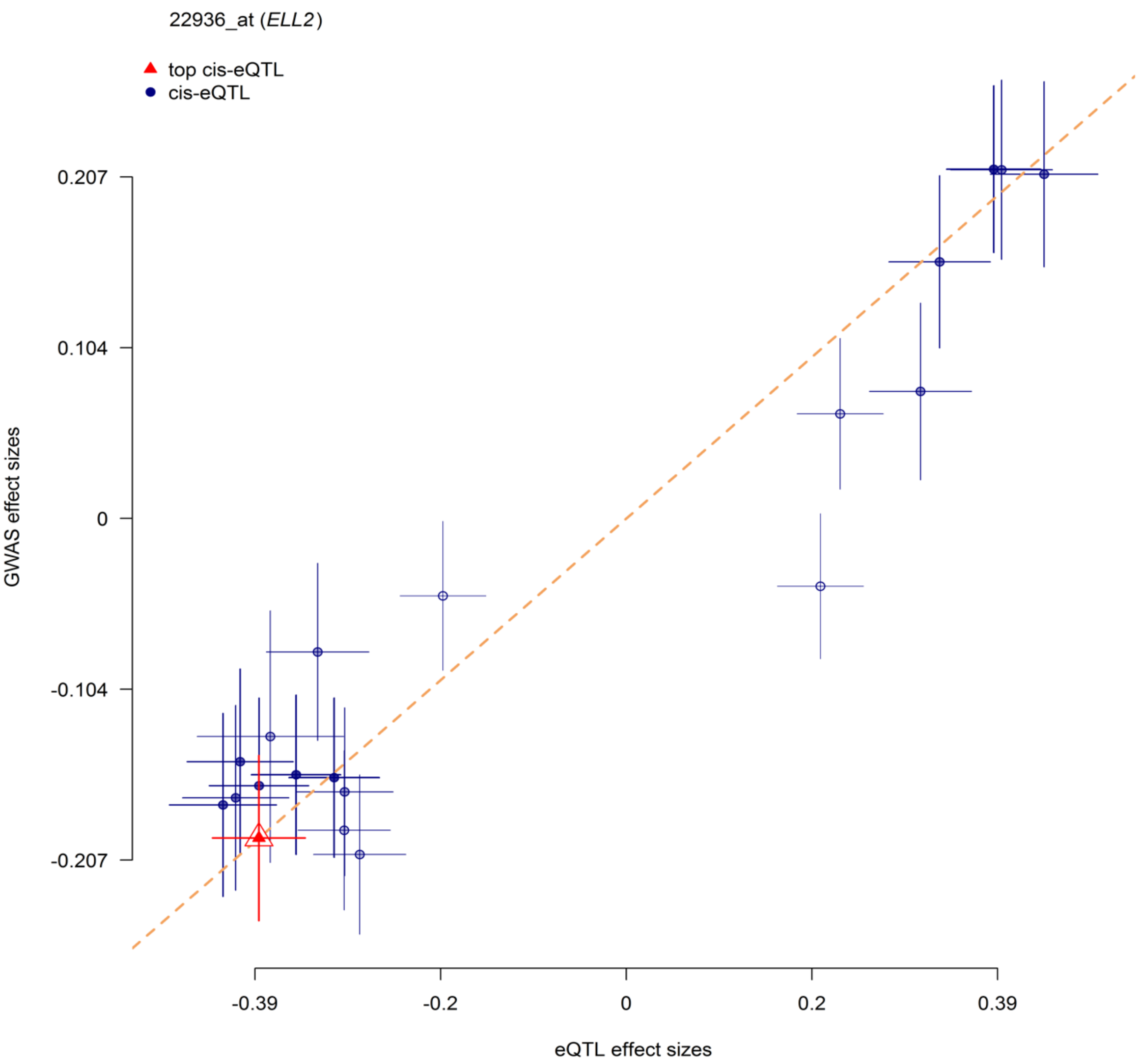

Figure S1, related to Figure 1. Effect sizes of SNPs (used for the HEIDI test) from GWAS meta-analysis plotted against those for SNPs from the respective eQTL study in SMR analysis. The orange dashed lines correspond to the estimate of b_{xy}, at the top *cis*-eQTL. Error bars correspond to standard errors of SNP effects.

Table S1, related to Figure 1. Coverage of sequence variation at 5q15 risk locus by imputation.

Germline variants within chr5:94,700,000-96,000,000 (number of variants (n) = 18,228) were defined by whole-genome sequencing (WGS) data of 640 MM patients sequenced by the CoMMpass Study. To calculate sequence variation coverage by the imputation methods used, biallelic variants from the WGS dataset with minor allele frequency (MAF) > 0.01 and 0.05 were compared with **(A)** the imputation panels (UK10K and 1000 Genome Project) and **(B)** the imputed UK/German GWAS datasets (INFO score > 0.8).

(A) Imputation reference panels (UK10K and 1000 Genome Project)

WGS (Total number of variants called = 18,228)	Coverage of sequence variation
MAF > 0.01, biallelic only (n=4,487)	94.8%
MAF > 0.05, biallelic only (n=2,437)	96.5%

(B) Imputed GWAS datasets (UK or German)

WGS (Total number of variants called = 18,228)	Coverage of sequence variation	
	UK	German
MAF > 0.01, biallelic only (n=4,487)	72.4%	70.3%
MAF > 0.05, biallelic only (n=2,437)	91.1%	90.6%

Table S2, related to Figure 1. Germline coding variants within *ELL2*.

Shown is the association of coding variants within *ELL2* with MM risk, assessed with 513 MM cases from the UK MyIX and MyXI clinical trials and 1,569 UK controls from the UK 1958 Birth Cohort (Scales et al., 2017). Position of SNP based on NCBI build 37 of the human genome. r^2 with respect to rs11372862. REF, reference allele; ALT, alternate allele with their allele counts in case and control populations. Also shown the MAF; minor allele frequency for cases and controls. *P*-value derived from Fisher's exact test (two-sided) assessing the difference in allele frequency in cases and controls. NA, SNP ID not available. Asterisk (*) denotes variants with no call rate of > 0.03 across either case or control samples.

					Cases			Controls			
**Variant**	**Position (bp)**	r^2	**REF**	**ALT**	**REF**	**ALT**	**MAF**	**REF**	**ALT**	**MAF**	***P*-value**
rs141910684	95234139	<0.001	G	A	819*	1*	0.0012	3086	2	0.00065	0.51
rs3777204	95234350	0.91	A	G	772	230	0.23	2187	849	0.28	1.76×10^{-3}
rs3777203	95234377	0.92	C	T	785	219	0.22	2110*	822*	0.28	9.71×10^{-5}
rs3777202	95234392	0.92	A	C	795	223	0.22	1953*	815*	0.29	3.34×10^{-6}
rs17085249/COSM1132083	95236415	0.92	G	A	788	210	0.21	2308	828	0.26	6.80×10^{-4}
rs41276265/COSM1620674	95236454	0.06	G	C	997	13	0.013	3080	56	0.018	0.32
rs3815768	95236459	0.92	C	T	791	213	0.21	2307	827	0.26	9.54×10^{-4}
rs113894818	95236474	0.007	G	T	986	14	0.014	3063	61	0.020	0.28
NA	95242281	<0.001	C	G	1021	1	0.00098	2228*	0*	0	0.31
NA	95242383	<0.001	T	C	1021	1	0.00098	3066	0	0	0.25
rs139040465	95249572	0.003	G	A	1022	2	0.0020	3119	7	0.0022	1.00
NA	95249586	<0.001	C	T	1025	1	0.00097	3090	0	0	0.25
rs146249322	95278729	0.02	T	C	990	4	0.0040	3111	23	0.0073	0.37

Table S3, related to Figure 1. Subtype case-control and case-only analyses at 5q15.

(A) Subtype case-control analysis for MM hyperdiploid (HRDMM) (1,363 cases, 7,304 controls) and for non-HRDMM cases (1,339 cases, 7,304 controls).

(B) Subtype case-only analysis for 1,363 HRDMM cases.

Position of SNP based on NCBI build 37 of the human genome. Odds ratio (OR) derived relative to the risk allele with 95% confidence interval (CI) shown. Meta *P*-value derived from meta-analysis of UK and German GWAS datasets. P_{het} represents Cochran's test of between-study heterogeneity *P*-value. I^2 denotes the proportion of the total variation due to heterogeneity, with values ≥75% considered to be characteristic of large heterogeneity.

(A) Case-control

SNP	Position (bp)	Risk allele	OR	95% CI	Meta *P*-value	P_{het}	I^2
**HRDMM**							
rs1423269	95255724	A	1.24	1.12-1.36	2.85×10^{-5}	0.86	0%
rs11372862	95257775	A	1.26	1.14-1.41	1.37×10^{-5}	0.52	0%
**Non-HRDMM**							
rs1423269	95255724	A	1.09	0.98-1.20	0.11	0.64	0%
rs11372862	95257775	A	1.12	1.00-1.25	0.05	0.88	0%

(B) Case-only

SNP	Position (bp)	Risk allele	OR	95% CI	Meta *P*-value	P_{het}	I^2
rs1423269	95255724	A	1.11	1.00-1.25	0.06	0.61	0%
rs11372862	95257775	A	1.13	1.00-1.28	0.04	0.37	0%

Table S4, related to Figure 2. SNPs within *ELL2* enhancer correlated with rs11372862 (r^2≥0.8).

Position of SNP based on NCBI build 37 of the human genome. r^2 with respect to rs11372862. Odds ratio (OR) derived relative to the risk allele with 95% confidence interval (CI) shown. Meta *P*-value derived from **(A)** meta-analysis of the UK MyIX and MyXI and German GMMG GWAS with 3,790 cases and 7,304 controls, **(B)** meta-analysis of HRDMM case-control analyses from the UK and German populations with 1,363 cases and 7,304 controls.

Emboldened are SNPs within interacting fragment with *ELL2* promoter (chr5:95,260,175-95,264,576; * in **Figure 2**). P_{het} represents Cochran's test of between-study heterogeneity *P*-value. I^2 denotes the proportion of the total variation due to heterogeneity, with values ≥75% considered to be characteristic of large heterogeneity.

(A) UK and German GWAS

SNP	Position (bp)	r^2	Risk allele	OR	95% CI	Meta *P*-value	P_{het}	I^2
rs3777185	95259862	0.97	C	1.15	1.07-1.22	6.11×10^{-5}	0.87	0%
**rs6877329**	**95261538**	**0.93**	**C**	**1.14**	**1.07-1.22**	**8.24×10^{-5}**	**0.62**	**0%**
**rs3777184**	**95262044**	**0.97**	**G**	**1.15**	**1.07-1.23**	**5.91×10^{-5}**	**0.88**	**0%**
rs889302	95264652	0.92	A	1.14	1.07-1.22	7.44×10^{-5}	0.65	0%
rs2015159	95265087	0.92	C	1.14	1.07-1.22	7.21×10^{-5}	0.65	0%
rs4563648	95265556	0.92	G	1.14	1.07-1.22	6.42×10^{-5}	0.65	0%

(B) UK and German HRDMM case-control

SNP	Position (bp)	r^2	Risk allele	OR	95% CI	Meta *P*-value	P_{het}	I^2
rs3777185	95259862	0.97	C	1.24	1.12-1.36	2.75×10^{-5}	0.88	0%
**rs6877329**	**95261538**	**0.93**	**C**	**1.22**	**1.10-1.34**	**9.65×10^{-5}**	**0.51**	**0%**
**rs3777184**	**95262044**	**0.97**	**G**	**1.24**	**1.12-1.37**	**2.38×10^{-5}**	**0.88**	**0%**
rs889302	95264652	0.92	A	1.22	1.10-1.34	8.73×10^{-5}	0.61	0%
rs2015159	95265087	0.92	C	1.22	1.10-1.34	8.16×10^{-5}	0.61	0%
rs4563648	95265556	0.92	G	1.22	1.11-1.35	7.00×10^{-5}	0.60	0%

Table S5, related to Figure 3 and 4. eQTL analyses.

(A) The relationship between rs1423269, rs11372862, rs6877329 genotypes and *ELL2* expression was assessed in hyperdiploid (HRDMM) and non-HRDMM CD138-selected MM plasma cells from 170 UK cases (GEO: GSE21349) and 602 German MM patients (EMBL-EBI: E-MTAB-2299). Association between SNP genotype and *ELL2* expression (22936_at) was evaluated based on the significance of linear regression coefficients. Meta *P*-value derived from meta-analysis of the UK and German datasets. Direction of eQTL is shown with respect to the risk alleles.

(B) The relationship between rs1423269, rs11372862, rs6877329 genotypes and genes with which *ELL2* is correlated in expression (*BiP*, *ATF6* and *POU2AF1* in **Figure 4**) was assessed in hyperdiploid (HRDMM) and non-HRDMM CD138-selected MM plasma cells from 170 UK cases (GEO: GSE21349) and 602 German MM patients (EMBL-EBI: E-MTAB-2299). *ELL1* expression data is not available as not expressed in MM patients ($\log_2$ expression < 3.5 in $\geq 95\%$ samples). Association between SNP genotype and gene expression was evaluated based on the significance of linear regression coefficients. Meta *P*-value derived from meta-analysis of the UK and German datasets.

(A)

SNP	Position (bp)	Risk allele	Meta *P*-value	Direction
**HRDMM**				
rs1423269	95255724	A	4.45×10^{-10}	Decrease
rs11372862	95257775	A	1.40×10^{-10}	Decrease
rs6877329	95261538	C	1.35×10^{-9}	Decrease
**Non-HRDMM**				
rs1423269	95255724	A	1.60×10^{-6}	Decrease
rs11372862	95257775	A	1.53×10^{-6}	Decrease
rs6877329	95261538	C	4.07×10^{-7}	Decrease

(B)

SNP	Position (bp)	Risk allele	Meta *P*-value		
			BiP (3309_at)	*ATF6* (22926_at)	*POU2AF1* (5450_at)
**HRDMM**					
rs1423269	95255724	A	0.17	0.95	0.39
rs11372862	95257775	A	0.78	0.72	0.57
rs6877329	95261538	C	0.28	1.00	0.48
**Non-HRDMM**					
rs1423269	95255724	A	0.65	0.38	0.63
rs11372862	95257775	A	0.94	0.18	0.69
rs6877329	95261538	C	0.60	0.57	0.55

Table S6, related to Figure 3. Investigating preferential amplification of the risk allele.

Number of HRDMM samples, heterozygous for the risk allele, with somatic amplification of the risk (AAB). *P*-value derived from exact binomial test of consensus.

	Inferred state		
	AAB	ABB	*P*-value
**MyXI Exomes**	34	23	0.18
**CoMMpass Exomes**	27	23	0.67

Table S7, related to Figure 4. Effect of rs6877329 genotypes and *ELL2*, *BiP*, *ATF6*, *ELL1* and *POU2AF1* expression on patient survival.

(A) Data from the UK-GWAS (1,165 cases from the UK MRC Myeloma IX trial, UK-MyIX; 877 cases from the UK MRC Myeloma XI trial, UK-MyXI) and the German-GWAS (511 cases from the GER-GMMG trial). Shown is the hazard ratio (HR) relative to the C-risk allele with associated 95% confidence intervals (CI).

Overall survival and progression-free survival analyses comparing patients with upper and lower quartile of **(B)** *ELL2*, **(C)** *BiP*, **(D)** *ATF6* and **(E)** *ELL1* and **(F)** *POU2AF1* expression. Data from 259 MM individuals from the UK MRC Myeloma IX trial (UK-MyIX; GEO: GSE21349) and 246 MM individuals from the German GMMG clinical trial (GER-GMMG; EMBL-EBI: E-MTAB-372) with expression data. Shown is the hazard ratio (HR) with associated 95% confidence intervals (CI).

(A) Relationship between rs6877329 genotypes and patient survival

Datasets		Overall survival	Progression-free survival
UK-MyIX	HR (95% CI)	0.95 (0.83-1.09)	0.98 (0.89-1.10)
	P-value	0.47	0.80
UK-MyXI	HR (95% CI)	1.00 (0.80-1.26)	1.03 (0.88-1.20)
	P-value	0.99	0.71
GER-GMMG	HR (95% CI)	0.96 (0.74-1.26)	0.96 (0.81-1.15)
	P-value	0.80	0.66

(B) Relationship between *ELL2* expression and patient survival

Datasets		Overall survival	Progression-free survival
UK-MyIX	HR (95% CI)	0.90 (0.59-1.37)	1.16 (0.81-1.67)
	P-value	0.62	0.41
GER-GMMG	HR (95% CI)	1.06 (0.55-2.07)	0.89 (0.56-1.42)
	P-value	0.85	0.63

(C) Relationship between *BiP* expression and patient survival

Datasets		Overall survival	Progression-free survival
UK-MyIX	HR (95% CI)	0.78 (0.52-1.17)	0.86 (0.60-1.23)
	P-value	0.23	0.41
GER-GMMG	HR (95% CI)	0.75 (0.40-1.42)	0.77 (0.49-1.21)
	P-value	0.38	0.25

(D) Relationship between *ATF6* expression and patient survival

Datasets		Overall survival	Progression-free survival
UK-MyIX	HR (95% CI)	0.89 (0.60-1.34)	0.84 (0.59-1.21)
	P-value	0.59	0.35
GER-GMMG	HR (95% CI)	1.09 (0.58-2.07)	1.02 (0.64-1.61)
	P-value	0.79	0.94

(E) Relationship between *ELL1* expression and patient survival

Datasets		Overall survival	Progression-free survival
UK-MyIX	HR (95% CI)	0.99 (0.66-1.48)	0.93 (0.65-1.32)
	P-value	0.96	0.68
GER-GMMG	HR (95% CI)	1.10 (0.57-2.15)	0.95 (0.59-1.53)
	P-value	0.77	0.82

(F) Relationship between *POU2AF1* expression and patient survival

Datasets		Overall survival	Progression-free survival
UK-MyIX	HR (95% CI)	1.14 (0.76-1.72)	1.25 (0.87-1.80)
	P-value	0.52	0.23
GER-GMMG	HR (95% CI)	1.02 (0.55-1.90)	1.25 (0.80-1.96)
	P-value	0.95	0.33

Supplemental Experimental Procedures

Ethics

Collection of patient samples and associated clinico-pathological information was undertaken with written informed consent and relevant ethical review board approval at respective study centers in accordance with the tenets of the Declaration of Helsinki. Specifically, the MRC Leukemia Data Monitoring and Ethics committee (MREC 02/8/95, ISRCTN68454111, MREC 17/09/09, ISRCTN49407852) and University of Heidelberg Ethical Commission (229/2003, S-337/2009, AFmu-119/2010).

Genome-wide association study data

The UK-GWAS and German-GWAS of MM have been previously reported (Mitchell et al., 2016). Briefly, the UK-GWAS comprised 2,329 MM cases, including 702 with HRDMM, genotyped using Illumina Human OmniExpress-12 v1.0 arrays (Illumina, San Diego). Cases were recruited through the UK MRC Myeloma-IX (MyIX) and Myeloma-XI (MyXI) trials (ISRCTN68454111: MyIX http://www.isrctn.com/search?q=ISRCTN68454111 and ISRCTN49407852: MyXI http://www.isrctn.com/search?q=ISRCTN49407852). Controls were provided by the Wellcome Trust Case Control Consortium 2 (http://www.wtccc.org.uk/) with 2,698 individuals in the 1958 British Birth Cohort genotyped using Hap1.2M-Duo Custom array data and 2,501 individuals from the UK Blood Service.

The German-GWAS comprised 1,512 MM cases, including 661 with HRDMM, genotyped using Illumina Human OmniExpress-12v1.0 arrays. Cases were recruited by the GMMG coordinated by the University Clinic, Heidelberg (ISRCTN06413384: GMMG-HD3 http://www.isrctn.com/search?q=ISRCTN06413384; ISRCTN64455289: GMMG-HD4 http://www.isrctn.com/search?q=ISRCTN64455289; and ISRCTN05745813: GMMG-HD5 http://www.isrctn.com/search?q=ISRCTN05745813). Controls comprised 2,107 healthy individuals from the Heinz Nixdorf Recall study genotyped using Illumina HumanOmni1-Quadv1 and HumanOmniExpress-12v1.0 arrays.

GWAS quality control has been described previously (Mitchell et al., 2016). To recover untyped genotypes we performed imputation using IMPUTE2 v2.3 with a combined UK10K and 1000 Genomes Project (phase 1 integrated release 3, March 2012) panel for reference (1000Genomes, 2010; Huang et al., 2015). Poorly imputed SNPs (INFO score < 0.80) were excluded. To assess sequence variation coverage by the GWAS arrays and current imputation methods we used WGS data from 640 MM patients from the CoMMpass Study (Craig et al., 2013). Genotypes of 18,228 variants called from the WGS data within chr5:94,700,000-96,000,000 were compared with the imputation reference panels used and the imputed genotypes from UK and German GWAS datasets.

Frequentist association testing between SNP genotype and MM was performed using logistic regression under an additive genetic model in SNPTESTv2.5 (Marchini et al., 2007). Meta-analysis was undertaken under a fixed-effects model using inverse variance weighting in META v1.7 (Marchini et al., 2007). To look for independent effects, conditional analysis was performed with SNPTESTv2.5 with genotypes from UK and German GWAS individuals conditioning on rs11376892. Association of germline coding variants within *ELL2* with MM risk was estimated using germline WES data from 513 MM patients and 1,569 controls as detailed in Scales et al., 2017. Fisher's exact test (two-sided) was used to assess the difference in allele frequency of the coding variants in cases and controls.

Subtype detection

Karotyping was used for cytogenetic studies of MM cells and standard criteria for the definition of a clone were applied. Fluorescence *in situ* hybridization (FISH) and ploidy classification of UK samples was conducted using the methodologies previously described (Chiecchio et al., 2006). FISH and ploidy classification of German samples was performed as previously described (Neben et al., 2010). Logistic regression in case-only and case-control analyses was used to assess tumor karyotype. Meta-analysis was undertaken under a fixed-effects model using inverse variance weighting in META v1.7 (Marchini et al., 2007).

Expression quantitative trait loci analysis

eQTL analyses were performed for CD138-purified plasma cells from 183 MRC MyIX trial patients (GEO: GSE21349) and 658 Heidelberg GMMG patients (EMBL-EBI: E-MTAB-2299) using Affymetrix Human Genome U133 2.0 Plus Array (Weinhold et al., 2015). Briefly, German and UK data were pre-processed separately, followed by analysis using a Bayesian approach to probabilistic estimation of expression residuals to infer broad variance components, accounting for hidden determinants influencing global expression. The association between genotype of SNPs and expression of genes within 500 kb either side of rs11372862 was evaluated based on the significance of linear regression coefficients. We pooled data from the two studies under a fixed-effects model. For subtype-specific eQTL, association analyses were performed between *ELL2* (22936_at), *BiP* (3309_at), *ATF6* (22926_at) and *POU2AF1* (5450_at) expression and SNPs rs11372862, rs1423269 or rs6877329 for 170 MRC MyIX trial patients and 602 Heidelberg GMMG patients with karyotype and expression data.

We carried out Summary data-based Mendelian randomization (SMR) analysis using previously established methods (Zhu et al., 2016). As previously advocated only probes with at least one eQTL *P*-value of $< 5.0 \times 10^{-8}$ were considered for SMR analysis. We set a threshold for the SMR test of *pSMR* < 0.00833 corresponding to a Bonferroni correction for 6 tests (6 probesets within 1 MB of rs11372862). We generated plots of the eQTL and GWAS associations at the locus, as well as plots of GWAS and eQTL effect sizes (i.e. corresponding to input for the HEIDI heterogeneity test). HEIDI test *P*-values < 0.05 were taken to indicate significant heterogeneity.

Cell lines

Human MM cell line KMS11 was obtained from the American Type Culture Collection (ATCC) and grown in Advanced RPMI 1640 Medium supplemented with GlutaMAX and 10% heat-inactivated fetal bovine serum (FBS). Human PCL cell lines L363 and JJN3 were obtained from Deutsche Sammlung von Mikroorganism und Zellkulturen GmbH (DSMZ). L363 was grown in Advanced RPMI 1640 supplemented with GlutaMAX and 15% heat-inactivated FBS. JJN3 was grown in 40% Dulbecco's MEM, 40% Iscove's MDM supplemented with 20% heat-inactivated FBS. Human lymphoblastoid cell line GM11992 were obtained from Coriell Institute and grown in RPMI 1640 culture medium supplemented with 15% FBS. Cell culture media were obtained from Life Technologies (Carlsbad, CA, USA) and FBS from PAA Laboratories (Pasching, Austria). Cells were cultured at 37°C, 100% relative humidity and 5% CO_2. Cell lines used were confirmed to be mycoplasma-free (PCR Mycoplasma Test Kit I/C, PromoCell, Heidelberg, Germany). Genotype of rs6877329 and rs3777184 in KMS11 and GM11992 was confirmed by Sanger sequencing using sequencing primers detailed below.

**Sequencing primers**	
rs3777184 F	GGAGTAGGAAGGGGAGGGTA
rs3777184 R	ATTGGCAGTTATCCCCAAAA
rs6877329 F	GAGGGGTTCTTTCCTCAAAT
rs6877329 R	CAAACTTACTGCAAACATTTAAACA

ENCODE and chromatin state dynamics

To explore the epigenetic profile of association signals at 5q15, we used DNAseI HS, transcription factor ChIP-seq data, histone modifications (H3K4Me1, H3K4Me3 and H3K27Ac) and ChromHMM in GM12878 from the ENCODE project (ENCODE, 2012). ChIP-seq on H3K4Me1, H3K4Me3 and H3K27Ac histone modifications were carried out in MM cell line KMS11 and PCL cell lines L363 and JJN3. Antibodies were obtained from Diagenode (Seraing, Belgium) for H3K4Me1 (C15410194), H3K4Me3 (C15410003) and H3K27Ac (C15410196) histone modifications. We used the *in silico* algorithms SIFT (Kumar et al., 2009) and PolyPhen-2 (Adzhubei et al., 2010) to predict the impact of amino acid substitutions.

Briefly, after cell lysing, sonication of nuclei was performed (BioRuptor; Diagenode, Liege, Belgium) to obtain 150-500 bp fragments. ChIP reactions were performed on a Diagenode SX-8G IP-Star Compact using Diagenode automated Ideal Kit reagents (C01010011, Diagenode). Protein A beads were incubated for 10 hours with 3-6 μg of

antibody and 2-4 million of sonicated cell lysate. ChIP samples were de-crosslinked at 65°C for 4 hours and subsequently treated for 30 minutes with RNAse Cocktail and Proteinase K. DNA was then purified (MiniElute PCR purification kit, Qiagen, Hilden, Germany), followed by library preparation according to manufacture (HTP Illumina library preparation kit, KAPA biosystem; Massachusetts, USA). Fourteen cycles of PCR were performed, followed by size selection for 200-400 bp fragments and final library purification (GeneRead Size Selection kit, Qiagen). ChIP libraries were sequenced using Illumina HiSeq 2000 (Illumina) with 100 bp single-ended reads. Generated raw reads were filtered for quality (Phred33 $\geq$ 30) and length (n $\geq$ 32), and adapter sequences were removed using Trimmomatic v0.22 (Lohse et al., 2012). Reads passing filters were then aligned to the human reference (hg19) using BWA v0.6.1. Peak calls are obtained using MACS2 v 2.0.10.07132012 (Feng et al., 2012) .

In situ promoter capture Hi-C

In situ promoter capture Hi-C was conducted in the cell line KMS11. *In situ* Hi-C libraries were prepared as previously described (Rao et al., 2014). To increase cell lysis efficiency, 3 aliquots of 8 million cells were fixed separately in 1% formaldehyde for 10 mins, each aliquot lysed in 15 mL lysis buffer and incubated on ice for 1 hour before being combined. Cross-linked DNA was digested by restriction enzyme *Hind*III (NEB, Ipswich, USA). Digested chromatin ends were filled and marked with biotin-14-dATP (ThermoFisher Scientific, Waltham, USA). The resulting blunt end fragments were ligated at 16°C in the nucleus with T4 DNA ligase (NEB) to minimize random ligation. DNA purified after crosslinking was reversed by proteinase K (Ambion, ThermoFisher Scientific) treatment. DNA was sheared by sonication (Covaris, Massachusetts, USA) and approximately 200-650 bp fragments selected. Biotin tagged DNA was pulled down with streptavidin beads and ligated with Illumina paired end adapters. Six cycles of PCR were performed to amplify libraries before capture. Promoter capture was based on 32,313 biotinylated 120-mer RNA baits (Agilent, Santa Clara, USA) targeting both ends of *Hind*III restriction fragments that overlap Ensembl promoters of protein-coding, non-coding, antisense, snRNA, miRNA and snoRNA transcripts. After library enrichment, a post-capture PCR step was carried out using 5 amplification cycles. Hi-C and CHi-C libraries were sequenced using Illumina HiSeq 2000 technology. Reads were aligned to the GRCh37 build using bowtie2 v2.2.6 and identification of valid di-tags was performed using HICUP v0.5.9. To declare significant contacts, HiCUP output was processed using CHiCAGO v1.1.8. Data from three independent biological replicates were combined to obtain a definitive set of contacts. The interaction within *ELL2* from its promoter with the highest score was plotted. Promoter capture Hi-C on GM12878 was obtained from publically available EMBL-EBI: E-MTAB-2323 (Mifsud et al., 2015).

Plasmid construction and luciferase assays

A 1 kb regulatory region with the risk alleles of rs6877329 and rs3777184 was amplified from human genomic DNA using primers detailed below, cloned into the PCR8/GW/TOPO vector and then transferred into pGL3 *luc+* SV40-promoter vector using Gateway technology (Life Technologies). The non-risk alleles of rs6877329 and rs3777184 were generated with site-directed mutagenesis using Quick Change XL (Agilent, Santa Clara, USA). Reporter constructs were introduced into KMS11 by nucleofection, using program X-01 on the Amaxa Nucleofector I (Amaxa Biosystems, Lonza, Basel, Switzerland). 5×10^{6} cells were resuspended in 100 µl Cell Line Nucleofector Solution V and mixed with 3 µg reporter plasmid DNA and 60 ng of internal control plasmid (pRL-SV40). Transiently transfected cells were grown for 24 hours before assaying with the Dual-Luciferase Reporter Assay System (Promega, Madison, USA) and the Fluoroskan Ascent FL plate reader (ThermoFisher Scientific). Relative luciferase activity was calculated as the ratio of luminescence from the experimental reporter to the internal control plasmid (pRL-SV40). Statistical significance was calculated using the two-tailed *t*-test over three biological replicates.

Plasmid construction primers	
ELL2 amplicon F	TAACTTTCTTTGCTGTGGGT
ELL2 amplicon R	GAATTTTGGGGATAACTGC
SDM rs6877329 F	CGGCATCCCAGGAAGGCAGTCCATCTCTTAA
SDM rs6877329 R	TTAAGAGATGGACTGCCTTCCTGGGATGCCG
SDM rs3777184 F	GGAAGGGCAGAGGGTACAGAGGATCCAACAC
SDM rs3777184 R	GTGTTGGATCCTCTGTACCCTCTGCCCTTCC
Sequencing F	TAACTTTCTTTGCTGTGGGT
Sequencing R	GAATTTTGGGGATAACTGC

Electrophoretic mobility shift assay

Nuclear protein was extracted from GM11992 cells using NE-PER nuclear and cytoplasmic extraction kit (ThermoFisher Scientific). Infrared dye DY-682-labeled (Eurofins Genomics, Germany) and unlabeled (Life Technologies) complementary oligonucleotides flanking rs6877329 were annealed to generate double-stranded EMSA probes detailed below. Each 20 µl binding reaction contained 50 fmol labeled target DNA, 1 × binding buffer (10 mM Tris, 50 mM KCl, 1 mM DTT [pH 7.5], 1 µg poly [dI.dC, Sigma-Aldrich], 2.5 mM DTT, and 10 µg nuclear protein extract). The reaction mix was incubated in the dark for 30 minutes at room temperature. Competition assays were performed by adding 100-fold molar excess of unlabeled probes. Super-shifts were performed by adding 2 µg CEBPβ antibody (sc-376591; Santa Cruz Biotechnology, Texas, USA) to the binding reaction and incubating for 15 minutes before the addition of labeled probe. DNA-protein complexes were resolved by electrophoresis on a 6% DNA retardation gel (Life Technologies) in 1× Tris-borate-EDTA (TBE) at 4 °C. Gels were imaged using the Odyssey Fc Infrared Imaging System (LI-COR Biosciences, Nebraska, USA).

EMSA oligos	
rs6877329 C F	CCCTCCGGCATCCCAGGAAGCCAGTCCATCTCTTAATTTTA
rs6877329 C R	TAAAATTAAGAGATGGACTGGCTTCCTGGGATGCCGGAGGG
rs6877329 G F	CCCTCCGGCATCCCAGGAAGGCAGTCCATCTCTTAATTTTA
rs6877329 G R	TAAAATTAAGAGATGGACTGCCTTCCTGGGATGCCGGAGGG

Gene expression analyses

We assessed the relationship between *ELL2* and *BiP*, *ATF6*, *POU2AF1*, *ELL1* gene expression (log_2-transformed) in two independent MM data sets GEO: GSE21349 (259 patients from the UK MRC MyIX clinical trial) and EMBL-EBI: E-MTAB-372 (246 patients from the German GMMG clinical trial) using Pearson's product-moment correlation test. Expression data generated using Affymetrix Human Genome U133 2.0 Plus Array. *P*-values from the two patient data sets were combined by Fisher's Method. Statistical tests were conducted using the R software version 3.1.3.

Relationship between SNP genotype and somatic copy number

To investigate whether the risk SNP rs6877329 was preferentially amplified in heterozygous individuals with trisomy of chromosome 5 we analyzed previously published WES data on 463 MyXI trial cases (Walker et al., 2015) in conjunction with WGS and WES data on 664 cases produced by the CoMMpass Study (Craig et al., 2013). Hyperdiploid cases were identified with chromosome 5 trisomy and amplification of at least one other odd numbered chromosome.

MyXI trial data: ExomeCNV was used to define amplification (logR > 0.1) (Sathirapongsasuti et al., 2011). Samples were identified as heterozygous for the risk SNP if heterozygous for five coding SNPs in LD with the risk variant (r^2 > 0.95) (rs17085249, rs3815768, rs3777202, rs3777203, rs3777204).

CoMMpass data: Amplification was defined using CBS (Venkatraman and Olshen, 2007) designated copy number segments called by CoMMpass (> 50% chromosome segment.mean > 0.3). WGS and WES reads were aligned to

GRCh37 build using BWA software (Li et al., 2009) and variants called according to GATK best practices (DePristo et al., 2011). As the whole genome sequencing data was low coverage, the germline genotype of the risk variant was determined using itself and an additional 18 SNPs in LD ($r^2 > 0.99$).

57 and 50 hyperdiploid samples heterozygous for the risk variant with chromosome 5 trisomy were identified in the MyXI and CoMMpass data sets respectively. Bam-readcounts (https://github.com/genome/bam-readcount/) was used to extract the counts of reads supporting the risk and non-risk variant from the WES data at 2/5 of the coding SNPs aforementioned, separated sufficiently to out rule common reads (rs3777204 and rs3815768, separated by > 1 kb). Given the counts of risk and non-risk reads sampled at these positions, each sample was assigned to its most probable state (amplification of the risk or non-risk allele) assuming a binomial distribution of counts. In germline heterozygote samples a bias towards the mapping of a reference read was observed. An adjustment to the probabilities was therefore made using the germline ratio of reference to alternate reads to estimate the mapping bias for each SNP.

Association between rs6877329 genotypes, *ELL2* expression and patient outcome

To examine the relationship between *ELL2*, *BiP*, *ATF6*, *ELL1* and *POU2AF1* expression and patient outcome, we used data from two independent patient cohorts (GEO: GSE21349, 259 patients from the UK MRC MyIX clinical trial; EMBL-EBI: E-MTAB-372, 246 patients from the German GMMG clinical trial). For each data set, patients were grouped by *ELL2*, *BiP*, *ATF6*, *ELL1* or *POU2AF1* expression (upper and lower quartiles). Analysis was performed using the log-rank test to estimate expression-associated hazard ratio and 95% confidence intervals. To examine the relationship between rs6877329 genotype and patient outcome, we analyzed GWAS data on the three patient cohorts specifically (i) 1,165 cases in the UK GWAS from the UK MRC MyIX trial; (ii) 877 MM cases in the UK GWAS from the UK MRC MyXI trial; (iii) 511 of the patients in the German GWAS from the German GMMG clinical trial. Analysis end points were myeloma-specific overall survival and progression-free survival, and analysis was performed as previously described (Johnson et al., 2016). Cox regression analysis was used to derive genotype-specific hazard ratio and associated 95% confidence intervals.

Supplemental References

1000Genomes (2010). A map of human genome variation from population-scale sequencing. Nature *467*, 1061-1073.

Adzhubei, I.A., Schmidt, S., Peshkin, L., Ramensky, V.E., Gerasimova, A., Bork, P., Kondrashov, A.S., and Sunyaev, S.R. (2010). A method and server for predicting damaging missense mutations. Nature methods *7*, 248-249.

Chiecchio, L., Protheroe, R.K., Ibrahim, A.H., Cheung, K.L., Rudduck, C., Dagrada, G.P., Cabanas, E.D., Parker, T., Nightingale, M., Wechalekar, A., *et al.* (2006). Deletion of chromosome 13 detected by conventional cytogenetics is a critical prognostic factor in myeloma. Leukemia *20*, 1610-1617.

Craig, D.W., Liang, W., Venkata, Y., Kurdoglu, A., Aldrich, J., Auclair, D., Allen, K., Harrison, B., Jewell, S., Kidd, P.G., *et al.* (2013). Interim Analysis Of The Mmrf Commpass Trial, a Longitudinal Study In Multiple Myeloma Relating Clinical Outcomes To Genomic and Immunophenotypic Profiles. Blood *122*, 532-532.

DePristo, M.A., Banks, E., Poplin, R., Garimella, K.V., Maguire, J.R., Hartl, C., Philippakis, A.A., del Angel, G., Rivas, M.A., Hanna, M., *et al.* (2011). A framework for variation discovery and genotyping using next-generation DNA sequencing data. Nature genetics *43*, 491-+.

ENCODE (2012). An integrated encyclopedia of DNA elements in the human genome. Nature *489*, 57-74.

Feng, J., Liu, T., Qin, B., Zhang, Y., and Liu, X.S. (2012). Identifying ChIP-seq enrichment using MACS. Nature protocols *7*, 1728-1740.

Huang, J., Howie, B., McCarthy, S., Memari, Y., Walter, K., Min, J.L., Danecek, P., Malerba, G., Trabetti, E., Zheng, H.F., *et al.* (2015). Improved imputation of low-frequency and rare variants using the UK10K haplotype reference panel. Nat Commun *6*, 8111.

Johnson, D.C., Weinhold, N., Mitchell, J.S., Chen, B., Kaiser, M., Begum, D.B., Hillengass, J., Bertsch, U., Gregory, W.A., and Cairns, D. (2016). Genome-wide association study identifies variation at 6q25.1 associated with survival in multiple myeloma. Nat Comm *7*, 10290.

Kumar, P., Henikoff, S., and Ng, P.C. (2009). Predicting the effects of coding non-synonymous variants on protein function using the SIFT algorithm. Nature protocols *4*, 1073-1081.

Li, H., Handsaker, B., Wysoker, A., Fennell, T., Ruan, J., Homer, N., Marth, G., Abecasis, G., Durbin, R., and Genome Project Data Processing, S. (2009). The Sequence Alignment/Map format and SAMtools. Bioinformatics *25*, 2078-2079.

Lohse, M., Bolger, A.M., Nagel, A., Fernie, A.R., Lunn, J.E., Stitt, M., and Usadel, B. (2012). RobiNA: a user-friendly, integrated software solution for RNA-Seq-based transcriptomics. Nucleic acids research *40*, W622-627.

Marchini, J., Howie, B., Myers, S., McVean, G., and Donnelly, P. (2007). A new multipoint method for genome-wide association studies by imputation of genotypes. Nature genetics *39*, 906-913.

Mifsud, B., Tavares-Cadete, F., Young, A.N., and Sugar, R. (2015). Mapping long-range promoter contacts in human cells with high-resolution capture Hi-C. *47*, 598-606.

Mitchell, J.S., Li, N., Weinhold, N., Forsti, A., Ali, M., van Duin, M., Thorleifsson, G., Johnson, D.C., Chen, B., Halvarsson, B.M., *et al.* (2016). Genome-wide association study identifies multiple susceptibility loci for multiple myeloma. Nat Commun *7*, 12050.

Neben, K., Jauch, A., Bertsch, U., Heiss, C., Hielscher, T., Seckinger, A., Mors, T., Muller, N.Z., Hillengass, J., Raab, M.S., *et al.* (2010). Combining information regarding chromosomal aberrations t(4;14) and del(17p13) with the International Staging System classification allows stratification of myeloma patients undergoing autologous stem cell transplantation. Haematologica *95*, 1150-1157.

Rao, S.S., Huntley, M.H., Durand, N.C., Stamenova, E.K., Bochkov, I.D., Robinson, J.T., Sanborn, A.L., Machol, I., Omer, A.D., Lander, E.S., *et al.* (2014). A 3D map of the human genome at kilobase resolution reveals principles of chromatin looping. Cell *159*, 1665-1680.

Sathirapongsasuti, J.F., Lee, H., Horst, B.A., Brunner, G., Cochran, A.J., Binder, S., Quackenbush, J., and Nelson, S.F. (2011). Exome sequencing-based copy-number variation and loss of heterozygosity detection: ExomeCNV. Bioinformatics *27*, 2648-2654.

Scales, M., Chubb, D., Dobbins, S.E., Johnson, D.C., Li, N., Sternberg, M.J., Weinhold, N., Stein, C., Jackson, G., Davies, F.E., *et al.* (2017). Search for rare protein altering variants influencing susceptibility to multiple myeloma. Oncotarget *8*, 36203-36210.

Venkatraman, E.S., and Olshen, A.B. (2007). A faster circular binary segmentation algorithm for the analysis of array CGH data. Bioinformatics *23*, 657-663.

Walker, B.A., Wardell, C.P., Murison, A., Boyle, E.M., Begum, D.B., Dahir, N.M., Proszek, P.Z., Melchor, L., Pawlyn, C., Kaiser, M.F., *et al.* (2015). APOBEC family mutational signatures are associated with poor prognosis translocations in multiple myeloma. Nat Commun *6*, 6997.
Weinhold, N., Meissner, T., Johnson, D.C., Seckinger, A., Moreaux, J., Forsti, A., Chen, B., Nickel, J., Chubb, D., Rawstron, A.C., *et al.* (2015). The 7p15.3 (rs4487645) association for multiple myeloma shows strong allele-specific regulation of the MYC-interacting gene CDCA7L in malignant plasma cells. Haematologica *100*, e110-113.
Zhu, Z., Zhang, F., Hu, H., Bakshi, A., Robinson, M.R., Powell, J.E., Montgomery, G.W., Goddard, M.E., Wray, N.R., Visscher, P.M., *et al.* (2016). Integration of summary data from GWAS and eQTL studies predicts complex trait gene targets. Nature genetics *48*, 481-487.
