# Supplementary material for: Genetic Predisposition to Multiple Myeloma at 5q15 Is Mediated by an ELL2 Enhancer Polymorphism
Source: Cell Rep. 2017 Sep 12;20(11):2556–64. doi: 10.1016/j.celrep.2017.08.062 (PMC5608969; doi:10.1016/j.celrep.2017.08.062)
Supplement: Document S2. Article plus Supplemental Information [file mmc2.pdf]

Cell Reports

Report

Genetic Predisposition to Multiple Myeloma at 5q15 Is Mediated by an *ELL2* Enhancer Polymorphism

Graphical Abstract

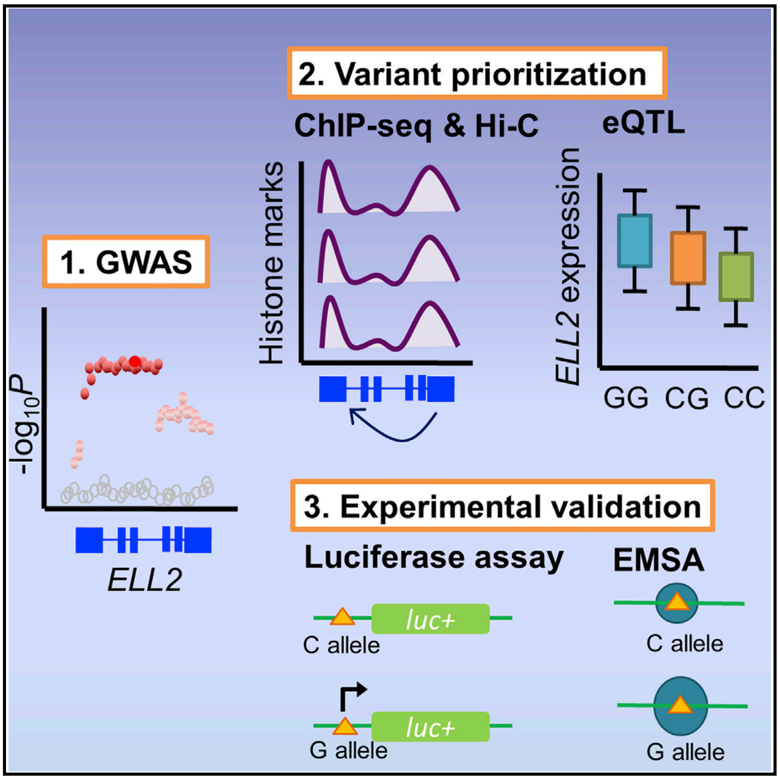

Highlights

- SNP rs6877329 underlies the association between 5q15 and multiple myeloma (MM)
- SNP rs6877329 forms a chromatin-looping interaction with the *ELL2* promoter
- rs6877329-C risk allele reduces enhancer activity in MM
- rs6877329-C confers lower *ELL2* expression in MM patients

Authors

Ni Li, David C. Johnson, Niels Weinhold, ..., Gareth J. Morgan, Martin Kaiser, Richard S. Houlston

Correspondence

richard.houlston@icr.ac.uk

In Brief

Li et al. find that rs6877329 underlies the 5q15 MM risk locus. Functional data demonstrate that rs6877329 resides within an enhancer that physically interacts with the *ELL2* promoter. The rs6877329-C risk allele reduces enhancer activity and is associated with reduced *ELL2* expression in MM patients.

CrossMark

Li et al., 2017, Cell Reports *20*, 2556–2564
September 12, 2017 © 2017 The Authors.
http://dx.doi.org/10.1016/j.celrep.2017.08.062

CellPress

OPEN ACCESS
CellPress

Cell Reports
Report

Genetic Predisposition to Multiple Myeloma at 5q15 Is Mediated by an *ELL2* Enhancer Polymorphism

Ni Li,[1,2] David C. Johnson,[2] Niels Weinhold,[3,4] Scott Kimber,[2] Sara E. Dobbins,[1] Jonathan S. Mitchell,[1] Ben Kinnersley,[1] Amit Sud,[1] Philip J. Law,[1] Giulia Orlando,[1] Matthew Scales,[1] Christopher P. Wardell,[3] Asta Försti,[8,9] Phuc H. Hoang,[1] Molly Went,[1] Amy Holroyd,[1] Fadi Hariri,[5] Tomi Pastinen,[5] Tobias Meissner,[6] Hartmut Goldschmidt,[4,7] Kari Hemminki,[8,9] Gareth J. Morgan,[3] Martin Kaiser,[2] and Richard S. Houlston[1,2,10,*]

[1]Division of Genetics and Epidemiology, The Institute of Cancer Research, Surrey SM2 5NG, UK
[2]Division of Molecular Pathology, The Institute of Cancer Research, Surrey SM2 5NG, UK
[3]Myeloma Institute for Research and Therapy, University of Arkansas for Medical Sciences, Little Rock, AK 72205, USA
[4]Department of Internal Medicine V, University of Heidelberg, 69117 Heidelberg, Germany
[5]McGill University and Genome Quebec Innovation Centre, Department of Human Genetics, McGill University, Montreal, Quebec, QC H3A 0G1, Canada
[6]Department of Molecular and Experimental Medicine, Avera Cancer Institute, La Jolla, CA 92037, USA
[7]National Centre of Tumor Diseases, 69120 Heidelberg, Germany
[8]German Cancer Research Center, 69120 Heidelberg, Germany
[9]Center for Primary Health Care Research, Lund University, 205 02 Malmo, Sweden
[10]Lead Contact
*Correspondence: richard.houlston@icr.ac.uk
http://dx.doi.org/10.1016/j.celrep.2017.08.062

SUMMARY

Multiple myeloma (MM) is a malignancy of plasma cells. Genome-wide association studies have shown that variation at 5q15 influences MM risk. Here, we have sought to decipher the causal variant at 5q15 and the mechanism by which it influences tumorigenesis. We show that rs6877329 G > C resides in a predicted enhancer element that physically interacts with the transcription start site of *ELL2*. The rs6877329-C risk allele is associated with reduced enhancer activity and lowered *ELL2* expression. Since *ELL2* is critical to the B cell differentiation process, reduced *ELL2* expression is consistent with inherited genetic variation contributing to arrest of plasma cell development, facilitating MM clonal expansion. These data provide evidence for a biological mechanism underlying a hereditary risk of MM at 5q15.

INTRODUCTION

Multiple myeloma (MM) is a malignancy of plasma cells primarily localized to the bone marrow (Kyle and Rajkumar, 2004; Kyle et al., 2007). The disease is genetically heterogeneous but can be broadly divided into hyperdiploid MM (HRDMM) and non-HRDMM subtypes (Gould et al., 1988; Sawyer et al., 1995; Smadja et al., 1998). Non-HRDMM is characterized by translocations of the immunoglobulin heavy chain (*IgH*) alleles at 14q32 with various recurrently observed genes, the significance of which is generally considered to be increased expression of the translocated partner gene. In contrast, HRDMM involves trisomies of odd-numbered chromosomes.

Although the etiological basis of MM is poorly understood, it has a significant genetic component as evidenced by a 2- to 4-fold increased risk in first-degree relatives of MM patients (Altieri et al., 2006; Landgren et al., 2009; Vachon et al., 2009). Our understanding of MM susceptibility has recently been transformed by genome-wide association studies (GWASs), which provide strong evidence that common genetic variation influences MM risk. So far, GWASs have identified 17 independent risk loci, with the signal annotating elongation factor for RNA polymerase II 2 (*ELL2*) at 5q15 being highly robust (Broderick et al., 2011; Chubb et al., 2015; Mitchell et al., 2016; Swaminathan et al., 2015). *ELL2* encodes a key component of the super-elongation complex (SEC) that drives secretory-specific immunoglobulin (Ig) mRNA production and transcriptional regulation in plasma cells (Park et al., 2014).

Here, we sought to identify the causal polymorphism(s) driving the 5q15 genetic association with MM susceptibility as a basis for understanding MM initiation. Our data are compatible with the rs6877329 variant as the functional basis of the 5q15 association, a genomic region, which through chromatin-looping interaction leads to the reduced expression of *ELL2*.

RESULTS

Fine Mapping and Epigenomic Profiling of the 5q15 Locus

We analyzed previously published UK and German GWAS datasets totaling 3,790 case subjects and 7,304 control subjects (Mitchell et al., 2016), genotyped on Illumina Human OmniExpress-12 v1.0, Illumina HumanOmni1-Quadv1, and Hap1.2M-Duo Custom arrays. To inform fine-mapping of the 5q15 risk locus, we imputed untyped genotypes in both GWASs using the UK10K (Huang et al., 2015) and 1000 Genomes Project

This is an open access article under the CC BY license (http://creativecommons.org/licenses/by/4.0/).

OPEN ACCESS
CellPress

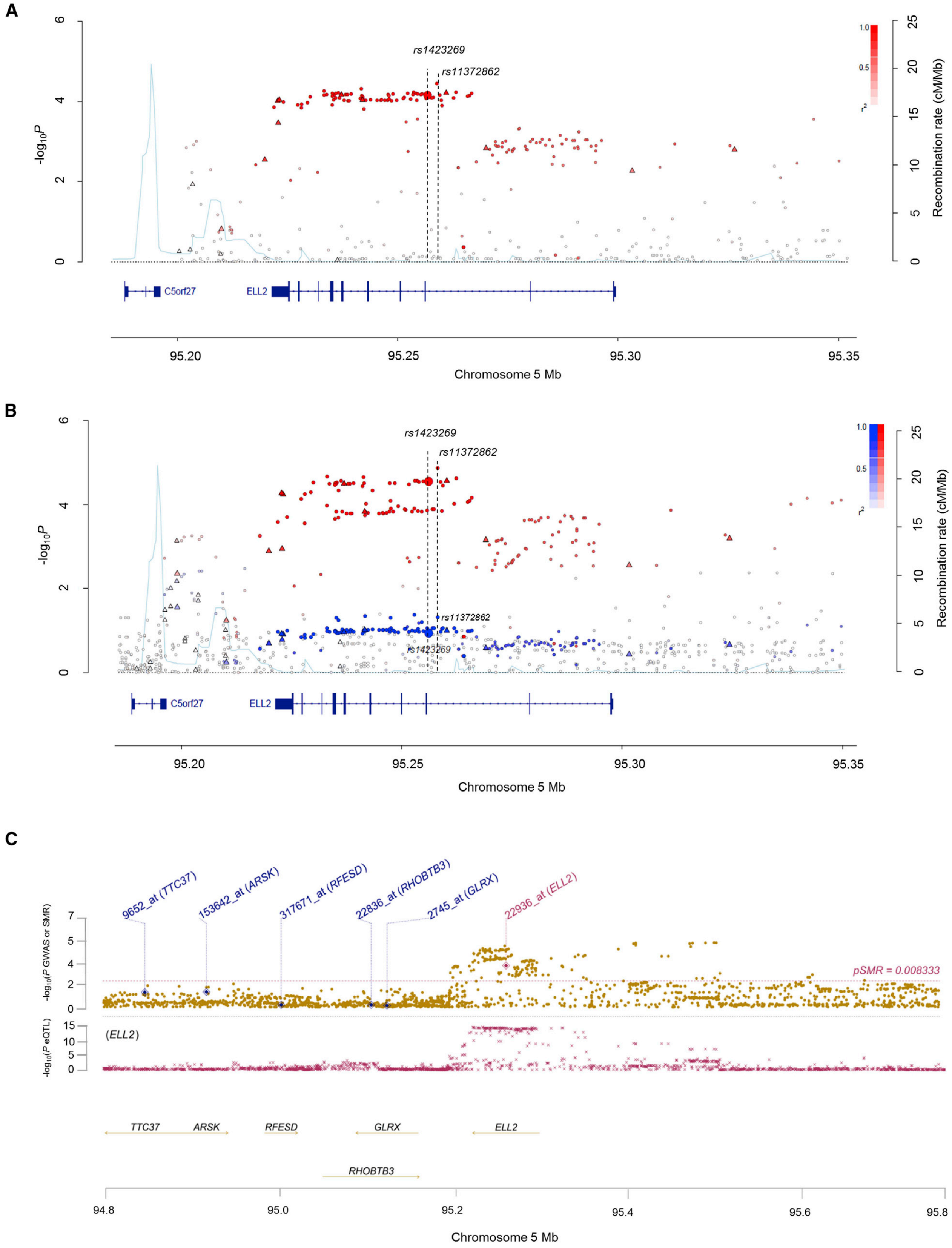

(legend on next page)

(The 1000 Genomes Project Consortium, 2010) as a reference. Overall, the strongest association across all forms of MM in a meta-analysis of the two GWAS datasets was provided by rs11372862 (OR = 1.16, $p = 3.75 \times 10^{-5}$, $P_{het} = 0.85$, Figure 1A), which is highly correlated with rs1423269 ($r^2 = 0.97$, $D' = 0.97$), the previously reported sentinel SNP for the 5q15 risk locus. Conditional analysis provided no evidence for additional independently associated SNPs at 5q15. By referencing germline whole-genome sequencing (WGS) data on 640 MM patients analyzed as part of the CoMMpass Study (Craig et al., 2013), we were able to confirm that the imputation captured >90% of sequence variation (minor allele frequency [MAF] > 0.05) within the linkage disequilibrium (LD) region encompassing rs11372862 (i.e., pairwise $r^2 \geq 0.1$) (Table S1). By analyzing the germline exomes of 513 MM case subjects from the UK Medical Research Council (MRC) MyIX and MyXI clinical trials and 1,569 UK control subjects from the UK 1958 Birth Cohort (Scales et al., 2017), we excluded the possibility that the 5q15 association signal is a consequence of LD with a rare disease-causing coding variant (Table S2).

Since there is previous evidence of subtype specificity for MM association (Weinhold et al., 2013), with the 11q13.3 association for MM being highly specific for t(11;14) MM, we examined whether the 5q15 association might also show evidence of subtype specificity. Stratifying MM by subtype revealed that risk at 5q15 was primarily associated with HRDMM (OR = 1.26, $p = 1.37 \times 10^{-5}$, $P_{\text{het}} = 0.52$) (Figure 1B; Table S3A). Case-only analysis also provided supportive evidence that the rs11372862 association was mainly driven by HRDMM (p = 0.04; Table S3B).

To gain further insight into the association, we performed an expression quantitative trait locus (eQTL) analysis using mRNA expression data on CD138-purified plasma cells from 841 MM case subjects from the UK MRC MyIX trial and German-Speaking Multiple Myeloma Multicenter Study Group (GMMG) trials. Specifically, we used Summary data-based Mendelian randomization (SMR) analysis to test for pleiotropy between GWAS signal and *cis*-eQTL for genes within 1 Mb of the lead SNP rs11372862 to identify a causal relationship (Zhu et al., 2016). eQTL analysis provided evidence for differential *ELL2* expression as being the basis of the 5q15 association (Figure 1C; Figure S1). Multiple SNPs (n = 90, including rs1423269, rs11372862, rs3777184, and rs6877329) mapping within *ELL2* in strong LD essentially defined a single haplotype defining MM risk and eQTL (Figure 1C).

The eQTL data suggest the 5q15 association with MM is likely to be mediated by the regulation of *ELL2* expression. To prioritize candidate risk variants, we examined the SNPs in LD ($r^2 \geq 0.8$) with rs11372862 within regulatory elements defined by B cell-specific DNase I hypersensitivity (DNaseI HS) and promoter/enhancer-associated histone marks (Figure 2). Six SNPs each correlated with rs11372862 localize within an 8 kb active enhancer region (Table S4), supported by ChromHMM, open chromatin analysis (DNaseI HS), as well as H3K4Me1, H3K4Me3, and H3K27Ac peaks in GM12878 (ENCODE Project Consortium, 2012), the MM cell line KMS11, and the plasma cell leukemia cell lines (PCL) JJN3 and L363.

Physical interactions between regulatory elements and promoters play a major role in regulating gene expression (Mifsud et al., 2015; Rao et al., 2014). Following our observation that six correlated SNPs localize within an enhancer element, we interrogated whether this genomic region physically interacts with the *ELL2* promoter in both KMS11 and GM12878 using promoter capture Hi-C (CHi-C) data. Within the active enhancer, rs6877329 and rs3777184 fall within an overlapping genomic fragment in both cell lines, forming a chromatin-looping interaction with the *ELL2* promoter (Figure 2; Table S4).

Effect of rs6877329 and rs3777184 Genotypes on Enhancer Activity

To measure the effect of rs6877329 and rs3777184 alleles on enhancer activity, we performed luciferase reporter assays in KMS11. Transfection with constructs containing the rs6877329-C risk allele displayed significantly lower normalized luminescence compared to non-risk G-allele construct (two-tailed t test p = 0.006, Figure 3A). rs3777184 genotype did not influence enhancer activity (two-tailed t test p = 0.57, Figure 3A). These data are thus consistent with a model of MM risk in which variation at rs6877329 is associated with decreased expression of *ELL2* (Table S5A). We next assayed protein-DNA interactions for rs6877329-C and rs6877329-G alleles using an electrophoretic mobility shift assay (EMSA). The G-allele formed stronger protein-DNA complexes compared with the C-allele, consistent with the region having differential transcription factor (TF) binding (Figure 3B). From ENCODE chromatin immunoprecipitation sequencing (ChIP-seq) data on GM12878, the CCAAT/enhancer-binding protein beta (CEBPB) is the only TF overlapping with rs6877329, albeit marginally. We did not, however, demonstrate an EMSA super-shift with CEBPB antibody with the G- and C-alleles (Figure 3B).

Figure 1. Regional Plots of Association Results of the 5q15 Locus

(A and B) The region of association maps to a ~40 kb haplotype block within *ELL2*. Genotyped (triangles) and imputed (dots) SNPs are shown based on their chromosomal position (NCBI build 37 human genome) on the x axis and $-\log_{10}$ p value on the y axis from (A) GWAS meta-analysis in UK MRC MyIX and MyXI trials and German GMMG trials (3,790 case subjects and 7,304 control subjects), and (B) HRDMM (red) and non-HRDMM (blue) case-control meta-analysis in the UK and German populations (1,363 HRDMM case subjects, 1,339 non-HRDMM case subjects, and 7,304 control subjects). Color intensity of each SNP reflects the extent of LD with the lead SNP, rs11372862 (white $r^2 = 0$ to dark red/blue $r^2 = 1$). Recombination rates, estimated using HapMap samples of European ancestry, are shown by a light blue line. The relative positions of *ELL2* and *C5orf27* mapping to 5q15 are shown. rs1423269 and rs11372862 are annotated in black dotted lines.

(C) Summary data-based Mendelian randomization analysis at 5q15. Upper panel - brown dots represent p values for SNPs from the HRDMM case-control meta-analysis (1,363 cases and 7,304 controls), diamonds represent p values for probes from the SMR test; lower panel - crosses represent eQTL p values of SNPs from MM plasma cells from 183 MRC MyIX trial patients (GEO: GSE21349) and 658 Heidelberg GMMG patients (EMBL-EBI: E-MTAB-2299), with genes passing the SMR (i.e., $P_{SMR} < 0.00833$) and HEIDI (i.e. $P_{HEIDI} > 0.05$) tests highlighted in red. Probeset ID refers to Affymetrix U133 2.0 Plus Array custom chip definition file (CDF v.17) mapping to Entrez genes.

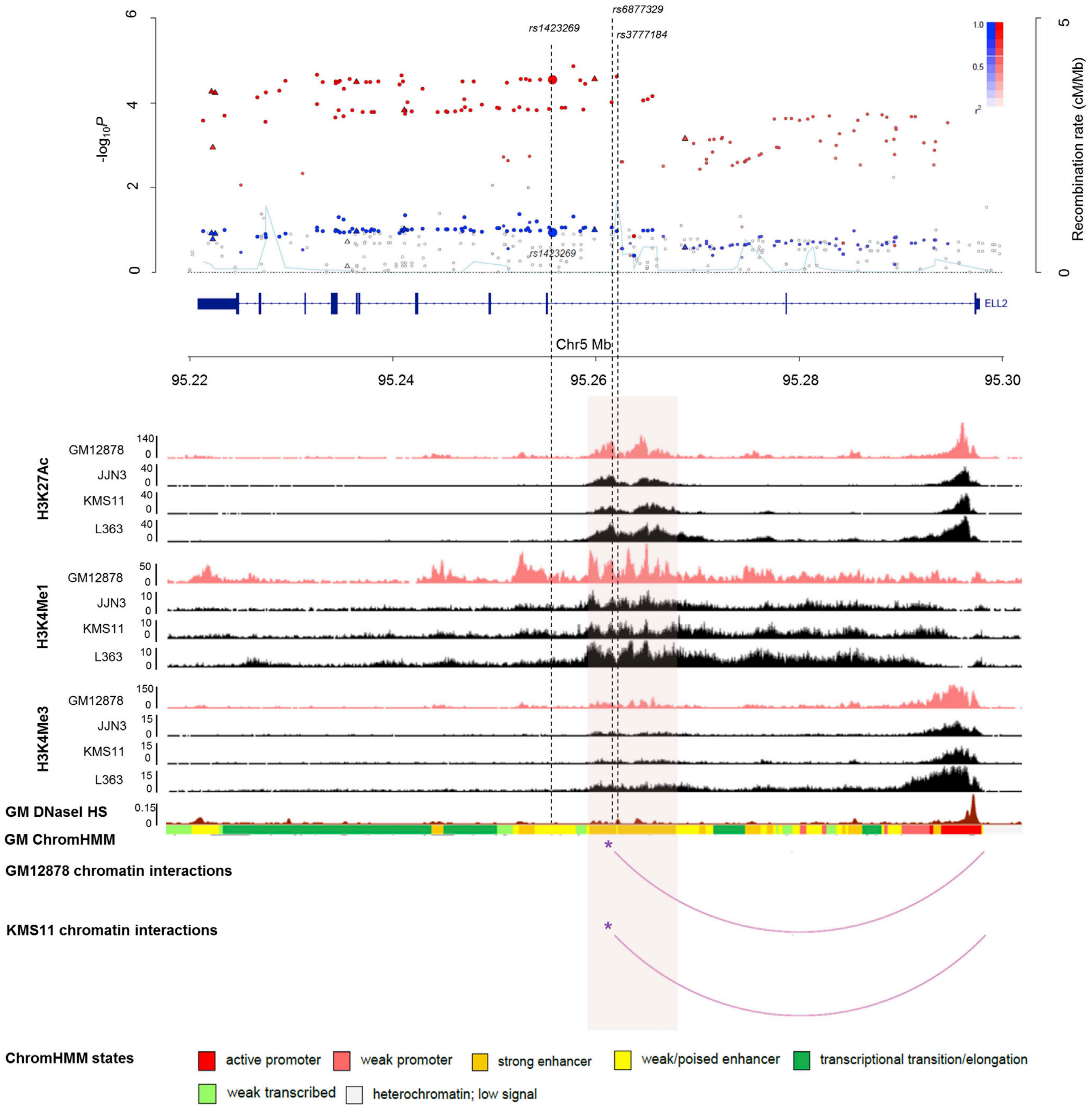

Figure 2. Epigenetic Landscape at the 5q15 Locus

HRDMM (red) and non-HRDMM (blue) case-control meta-analysis as shown in Figure 1B. ChIP-seq from GM12878 (pink peaks), JJN3, KMS11, and L363 (black peaks) are shown, annotated with the ChIP'd histone modification marks H3K4Me1, H3K4Me3, and H3K27Ac. DNaseI HS and ChromHMM data for GM12878 were assessed from ENCODE. A ~8kb active enhancer (chr5:95,259,093-95,267,656) within *ELL2* is shaded, predicted by ChromHMM, DNaseI HS, as well as H3K4Me1, H3K4Me3, and H3K27Ac peaks. rs3777185, rs6877329, rs3777184, rs889302, rs2015159, and rs4563648 are localized within the enhancer with $r^2 \geq 0.8$ with rs11372862 (Table S3). Asterisk (*) marks the enhancer region interacting with the *ELL2* promoter in both GM12878 and KMS11 (chr5:95,260,175-95,264,576) encompassing rs6877329 and rs3777184. The positions of rs1423269, rs6877329, and rs3777184 are marked with black dotted lines.

rs6877329 Risk Allele Is Not Preferentially Amplified in Hyperdiploid Myeloma

Trisomy of chromosome 5 typifies HRDMM. To investigate a possible relationship between heritable risk associated with rs6877329 and somatic mutation, we sought to determine whether the risk C-allele, associated with reduced *ELL2* expression, is preferentially amplified in HRDMM. We analyzed whole-exome sequencing (WES) data on two independent series of

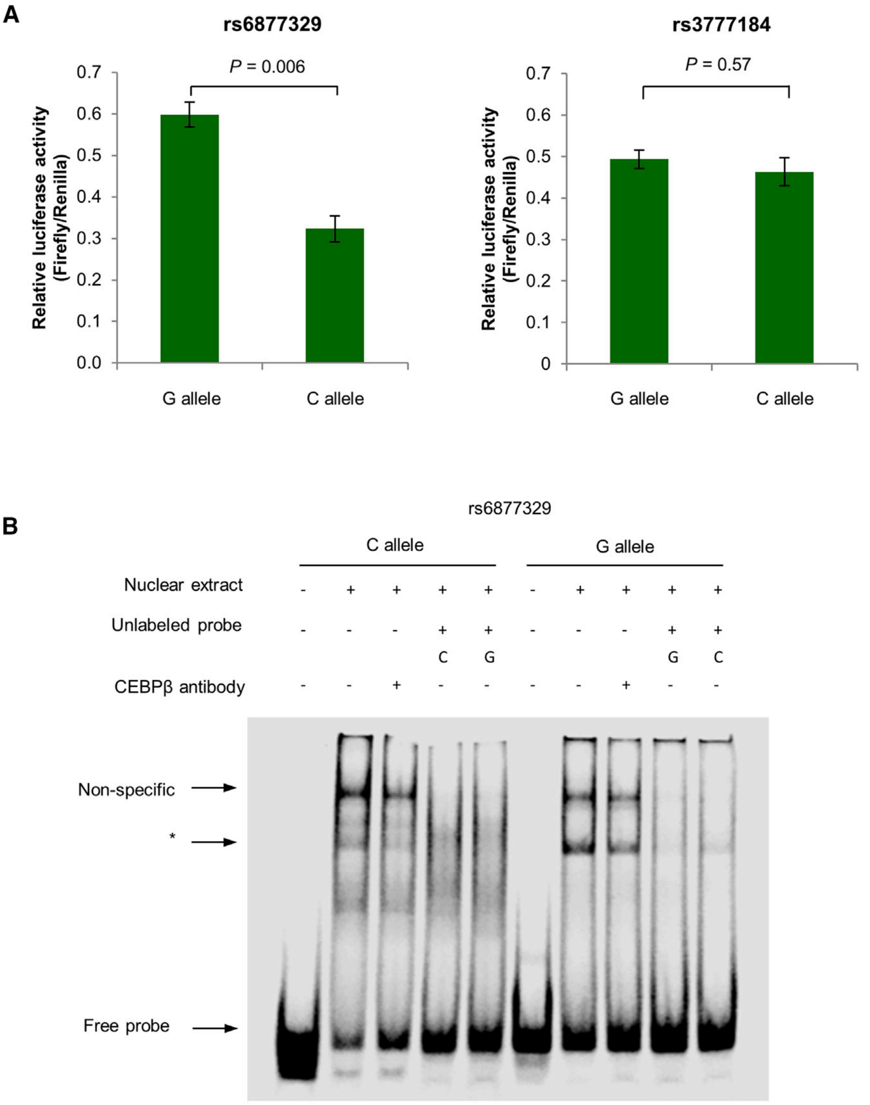

Figure 3. rs6877329 Is Associated with Reduced Enhancer Activity and Differential Nuclear Protein Binding

(A) rs6877329-C risk allele shows decreased expression over the protective G-allele. Allele-specific constructs containing a putative regulatory sequence flanking rs6877329 and rs3777184 were cloned into the pGL3 *luc*+ SV40-promoter vector and transfected into KMS11. The ratio of luminescence from the experimental pGL3-constructs to the Renilla internal control, pRL-SV40, was normalized to the empty pGL3 *luc*+ SV40-promoter vector. Data shown are mean ± SEM from three independent experiments performed in triplicate. Differences in gene expression were assessed by the two-tailed t test.

(B) Electrophoretic mobility shift assay in GM11992 showing differential nuclear protein binding to alleles of rs6877329. Increased protein binding observed for the non-risk G-allele. Asterisk (*) indicates specific protein shift band.

HRDMM tumors that were trisomic for chromosome 5 and heterozygous for rs6877329, using the highly correlated synonymous SNP rs3777204 and the missense SNP rs3815768 as proxies (rs3777204 r^2 = 0.99, *D'* = 1.00; rs3815768 r^2 = 0.95, *D'* = 0.99). rs3815768 is predicted to be benign and tolerated by PolyPhen-2 and SIFT, respectively. After correcting for the germline ratio of reference to alternate alleles in both series, we found no evidence of preferential duplication of the risk C-allele (Table S6).

ELL2 Expression Correlates with Unfolded Protein Response and SEC Components

We sought to establish a possible functional consequence of reduced *ELL2* expression on MM oncogenesis. A recent study of conditional knockout (cKO) *ELL2* mice identified 10 genes where loss of *ELL2* expression resulted in at least a 2-fold difference in expression (Park et al., 2014). We assessed mRNA expression correlation between *ELL2* and these 10 genes in the tumors from 505 MM patients (GEO: GSE21349 from the UK MRC MyIX trial; EMBL-EBI: E-MTAB-372 from the German GMMG trial). Although there was no evidence for a *trans*-eQTL in *BiP* (binding immunoglobulin protein), *ATF6* (activating TF 6), *ELL1* (elongation factor for RNA polymerase II, 1), and *POU2AF1* (POU class 2 associating factor 1) (Table S5B), these genes consistently correlated with *ELL2* expression in MM (Figure 4).

Impact of rs6877329 Genotype and Gene Expression on Patient Prognosis

To examine the relationship between rs6877329 genotype and *ELL2* expression on patient outcome, defined by overall survival (OS) and progression-free survival (PFS), we made use of data from the UK MRC MyIX and MyXI trials and the German GMMG trial, totaling 505 MM patients with expression data and 2,553 patients with genotype data. Meta-analysis of these data provided no evidence for an association between either rs6877329 genotype or the level of *ELL2* expression with either OS or PFS ($p > 0.4$; Tables S7A and S7B). We also found no evidence linking *BiP*, *ATF6*, *ELL1,* and *POU2AF1* expression to patient outcome ($p > 0.2$; Tables S7C–S7F).

DISCUSSION

We acknowledge that the large LD block at 5q15 presents limitations in prioritizing the functional variant underscoring the association. However, collectively our data demonstrate a plausible mechanism underlying MM risk being mediated through rs6877329, compatible with a differential effect on TF binding. Moreover, data are compatible with the rs6877329-C allele

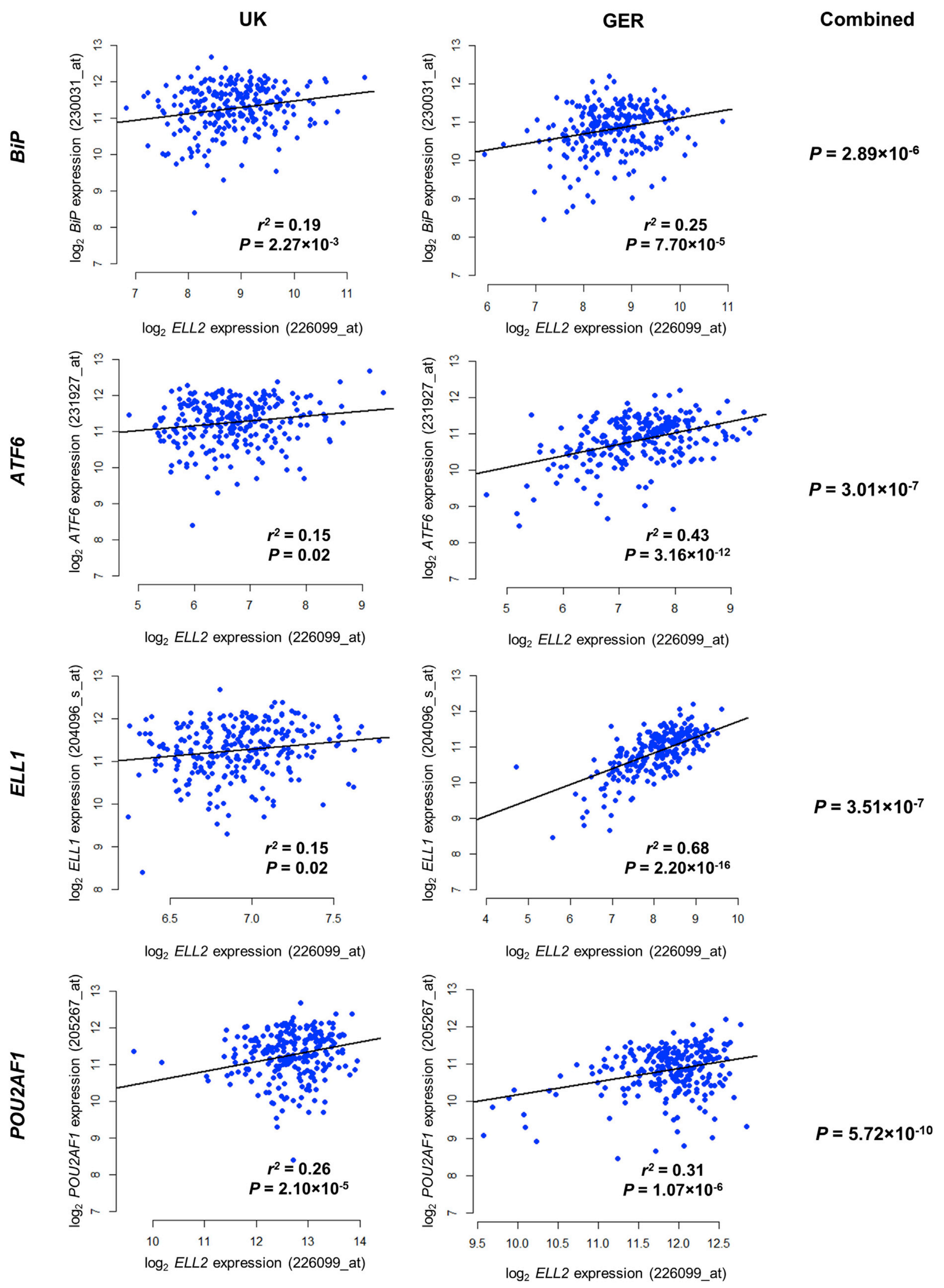

(legend on next page)

conferring increased risk through reduced expression of *ELL2*. Epigenetic and chromosome conformation capture data are consistent with rs6877329 localizing within a chromatin contact domain and overlapping a B cell enhancer. This interval forms a "loop domain," bringing it into physical contact close to the transcription start site of *ELL2*, separated by a linear distance of around 35 kb.

ELL2 plays a key role in the differentiation of mature B cells into plasma cells (Park et al., 2014). *ELL2* is induced 6-fold in plasma cells and drives secretory-specific *IgH* mRNA production via enhanced exon skipping and polyadenylation (Martincic et al., 2009). B cell lineage *ELL2* cKO mice show reduced numbers of plasma cells and a paucity of secreted Ig (Park et al., 2014). We have shown that individuals carrying the rs6877329-C risk allele have reduced *ELL2* transcript levels. The observation that the MM risk locus at 5q15 is associated with reduced levels of IgA and IgG in healthy individuals is thus consistent with a hypomorphic effect associated with reduced *ELL2* expression (Swaminathan et al., 2015). These data do not lend themselves to an obvious basis for allele-specific reduction in *ELL2* being associated with increased MM risk.

We observed a strong relationship between *ELL2* with *BiP* and *ATF6* expression in MM. *BiP* is a regulator of the UPR pathway during endoplasmic reticulum (ER) stress—a pathway heavily relied on by MM plasma cells for survival due to its active production and secretion of immunoglobulins (Vincenz et al., 2013). *BiP* has been associated with the activation of UPR inducers, such as *ATF6*, *PERK* (PKR-like ER kinase), and *IRE1* (inositol-requiring enzyme 1) (Bertolotti et al., 2000). Furthermore, the UPR pathway regulates the equilibrium between proliferation and cell death during ER stress, and the formation of autophagosome can be inhibited by *BiP* knockdown (Li et al., 2008). Collectively, these data are compatible with *ELL2* having a role in the UPR and autophagy regulation in MM through its interaction with *BiP* and *ATF6*. Importantly, due to the pre-existing ER stress in MM plasma cells, such cells are particularly prone to drug-induced ER stress by, for example, the proteasome inhibitor bortezomib (Obeng et al., 2006). *ELL2* is also associated with *ELL1* and *POU2AF1* expression in MM patients, the latter being a transcriptional coactivator of *OCT2* (octamer-binding protein 2), expression of which is required for B cell differentiation (Hodson et al., 2016). Reduced *ELL2* expression associated with 5q15 thus potentially suggests an impairment of SEC function and hindrance in plasma cell development.

We did not observe an association between rs6877329 genotype or *ELL2* expression level on patient survival in MM. While our analysis had 80% power to demonstrate a 10% difference in patient outcome at $p = 0.05$, we acknowledge that to detect a smaller impact would require much larger patient cohorts. Accepting this caveat, our findings are consistent with differential expression of *ELL2* being important in the early phase of MM tumor development rather than disease progression per se.

To directly inform of a possible relationship between the rs6877329-C risk allele and somatic mutation, we exploited the fact that the 5q15 association was primarily shown for HRDMM. The effects of chromosome 5 gain are likely driven by a selective advantage conferred by increased dosage of additional unknown factors unrelated to the primary function of *ELL2*. Evidence that HRDMM heterozygotes preferentially duplicate the chromosome 5 homolog with the rs6877329-C risk allele would have potentially suggested increased dosage of chromosome 5 gene expression relative to *ELL2*. Irrespective of a failure to demonstrate such a relationship and although speculative, it is possible that decreased *ELL2* expression would increase the probability of arrest of normal plasma cell development, facilitating MM clonal expansion. Since the association between rs6877329-C risk allele and *ELL2* expression is not exclusive to HRDMM, these data suggest that loss of *ELL2* activity is relevant in the context of both HRDMM and non-HRDMM primary initiating events.

In conclusion, we have shown reduced *ELL2* expression in MM patients carrying the rs6877329-C risk allele, thus providing a mechanistic basis for the 5q15 risk association for MM. Further functional studies, however, are required to fully decipher the biological basis of differential *ELL2* expression on MM oncogenesis.

EXPERIMENTAL PROCEDURES

Ethics

Collection of patient samples and associated clinico-pathological information was undertaken with written informed consent and relevant ethical review board approval at respective study centers in accordance with the tenets of the Declaration of Helsinki, specifically, the MRC Leukemia Data Monitoring and Ethics Committee (MREC 02/8/95, ISRCTN68454111, MREC 17/09/09, and ISRCTN49407852) and the University of Heidelberg Ethical Commission (229/2003, S-337/2009, AFmu-119/2010).

GWAS Data

The UK-GWAS and German-GWAS of MM have been previously reported (Mitchell et al., 2016). The diagnosis of MM (ICD-10 C90.0) was established in accordance with World Health Organization guidelines. All samples from patients for genotyping were obtained before treatment or at presentation. The UK-GWAS comprised 2,329 MM case subjects (1,060 male; mean age at diagnosis: 64.0 years), including 702 with HRDMM, recruited through the UK MRC MyIX and MyXI trials. Control subjects were provided by the Wellcome Trust Case Control Consortium 2 with 2,698 individuals in the 1958 British Birth Cohort and 2,501 individuals from the UK Blood Service. The German-GWAS comprised 1,512 MM case subjects (867 male; mean age at diagnosis: 59 years), including 661 with HRDMM. Case subjects were recruited by the GMMG trial. Control subjects comprised 2,107 healthy individuals from the Heinz Nixdorf Recall study. To recover untyped genotypes, we performed imputation using IMPUTE2 v2.3 with a combined UK10K and 1000 Genomes Project (phase 1 integrated release 3, March 2012) panel for reference (The 1000 Genomes Project Consortium, 2010; Huang et al., 2015). Poorly imputed SNPs (INFO score < 0.80) were excluded. Frequentist association testing between SNP genotype and MM was performed using logistic regression under an additive genetic model in SNPTESTv2.5 (Marchini et al., 2007). Meta-analysis was undertaken under a fixed-effects model using

Figure 4. Gene Expression Correlations with *ELL2* in Multiple Myeloma
Expression correlation between *ELL2* and *BiP,* and *ATF6, ELL1,* and *POU2AF1* was examined in 259 UK MM patients (GEO: GSE21349) and 246 German (GER) MM patients (EMBL-EBI: E-MTAB-372). Lines show linear regression fits. Expression correlation was assessed by Pearson's product-moment correlation test. p values were combined from independent datasets using Fisher's Method.

inverse variance weighting in METAv1.7 (Marchini et al., 2007). To look for independent effects, we performed conditional analysis with SNPTESTv2.5 with genotypes from UK and German GWAS individuals conditioning on rs11376892. Logistic regression in case-only and case-control analyses was used to assess tumor subtype.

Expression Quantitative Trait Loci Analysis

eQTL analyses were performed for genes and SNPs within 1 MB of rs11372862 for CD138-purified plasma cells from 183 UK MyIX trial patients and 658 German GMMG patients. Briefly, German and UK data were pre-processed separately, followed by analysis using a Bayesian approach to probabilistic estimation of expression residuals to infer broad variance components, accounting for hidden determinants influencing global expression. The association between genotype of SNPs and expression of genes within 500 kb either side of rs11372862 was evaluated based on the significance of linear regression coefficients. We pooled data from the two studies under a fixed-effects model. Subtype-specific eQTL analyses were performed for *ELL2, BiP, ATF6,* and *POU2AF1* expression and rs11372862, rs1423269, and rs6877329 for 170 UK MyIX trial and 602 German GMMG patients with subtype and expression data. We carried out SMR analysis using previously established methods (Zhu et al., 2016), with a threshold for the SMR test set at $P_{SMR} < 0.00833$ corresponding to Bonferroni correction.

ENCODE and Chromatin State Dynamics

To explore the epigenetic profile of association signals at 5q15, we used DNaseI HS, TF ChIP-seq data, histone modifications (H3K4Me1, H3K4Me3, and H3K27Ac), and ChromHMM in GM12878 from the ENCODE project (ENCODE Project Consortium, 2012). ChIP-seq on H3K4Me1, H3K4Me3, and H3K27Ac were carried out in KMS11, L363, and JJN3 cell lines.

In Situ Promoter Capture Hi-C

In situ promoter capture Hi-C libraries were prepared for KMS11 as previously described (Rao et al., 2014). The interaction within *ELL2* from its promoter with the highest score was plotted. Promoter capture Hi-C on GM12878 was obtained from EMBL-EBI: E-MTAB-2323 (Mifsud et al., 2015).

Plasmid Construction and Luciferase Assays

Regulatory region with rs6877329 and rs3777184 risk/non-risk alleles was cloned into pGL3 *luc*+ SV40-promoter vector (Promega, Madison, WI, USA). Reporter constructs were introduced into KMS11. Relative luciferase activity was calculated as the ratio of luminescence from the experimental reporter to the internal control plasmid (pRL-SV40). We calculated statistical significance by using the two-tailed t test over three biological replicates.

Electrophoretic Mobility Shift Assay

Nuclear protein from GM11992 cells was incubated with infrared dye DY-682-labeled double-stranded EMSA probes flanking rs6877329 (Eurofins Genomics, Germany) (Supplemental Experimental Procedures). Competition assays were performed by adding 100-fold molar excess of unlabeled probes. Super-shifts were performed by adding 2 μg CEBPB antibody (sc-376591; Santa Cruz Biotechnology, Texas, USA). DNA-protein complexes were resolved by electrophoresis on a 6% DNA retardation gel (Life Technologies, Carlsbad, CA, USA) in 1 × Tris-borate-EDTA (TBE).

Gene Expression Analyses

We assessed the relationship between *ELL2* and *BiP*, *ATF6*, *POU2AF1*, and *ELL1* gene expression ($\log_2$-transformed) in 259 UK MyIX clinical trial patients and 246 German GMMG clinical trial patients using Pearson's product-moment correlation test. p values from the two patient datasets were combined by Fisher's Method. We conducted statistical tests using the R software version 3.1.3.

Relationship between SNP Genotype and Somatic Copy Number

To investigate whether rs6877329 was preferentially amplified in heterozygous individuals with chromosome 5 trisomy, we analyzed WES data on 463 UK MyXI trial cases (Walker et al., 2015) in conjunction with WGS and WES data on 664 cases produced by the MM CoMMpass Study (Craig et al., 2013). 57 and 50 hyperdiploid samples heterozygous for the risk variant with chromosome 5 trisomy were identified in the MyXI and CoMMpass datasets, respectively. Given the counts of risk and non-risk reads sampled at two proxy coding SNPs (rs3777204 and rs3815768, $r^2 > 0.95$), each sample was assigned to its most probable state (amplification of the risk or non-risk allele) assuming a binomial distribution of counts and adjusting for reference mapping bias using germline read counts.

Association between rs6877329 Genotypes, *ELL2* Expression, and Patient Outcome

The relationship between *ELL2, BiP, ATF6, ELL1,* and *POU2AF1* expression and patient outcome (OS and PFS) was assessed with 259 UK MyIX clinical trial patients and 246 German GMMG clinical trial individuals grouped by their *ELL2* expression (upper and lower quartiles). Analysis was performed using the log-rank test to estimate expression-associated hazard ratio and the 95% confidence interval. Statistical tests were conducted using the R software version 3.1.3. We assessed the relationship between rs6877329 genotype and patient outcome using GWAS data on (1) 1,165 patients in the UK GWAS from the MyIX trial, (2) 877 MM patients in the UK GWAS from the MyXI trial, and (3) 511 of the patients in the German GWAS from the GMMG clinical trial. Cox regression analysis was used to derive genotype-specific hazard ratio and associated 95% confidence intervals.

DATA AND SOFTWARE AVAILABILITY

The accession number for the ChIP-seq data reported in this paper is EGA: S00001002414. The accession number for the Hi-C data reported in this paper is EGA: S00001002614.

SUPPLEMENTAL INFORMATION

Supplemental Information includes Supplemental Experimental Procedures, one figure, and seven tables and can be found with this article online at http://dx.doi.org/10.1016/j.celrep.2017.08.062.

AUTHOR CONTRIBUTIONS

N.L. and R.S.H. drafted the manuscript; M.K. contributed. N.L. and R.S.H. designed the study. N.L., S.K., G.O., and A.H. performed laboratory work. F.H. performed ChIP-seq experiments. H.G., G.J.M., K.H., and M.K. performed sample ascertainment and provided data. N.L., D.C.J., N.W., S.E.D., J.S.M., A.S., P.J.L., B.K., M.S., C.P.W., A.F., P.H.H., M.W., and T.M. performed bioinformatics and statistical analyses. All authors contributed to the final manuscript.

ACKNOWLEDGMENTS

Funding was provided by Myeloma UK, Bloodwise (13027), and Cancer Research UK (grant C1298/A8362 supported by the Bobby Moore Fund), the Rosetrees Trust, the Dietmar Hopp Foundation, and the German Ministry of Education and Science (grant BMBF: CLIOMMICS 01ZX1309). This study made use of genotyping data on the 1958 Birth Cohort generated by the Wellcome Trust Sanger Institute (http://www.wtccc.org.uk). The German study was supported by the Dietmar Hopp Stiftung, Germany; the German Cancer Aid (110,131); the German Ministry of Education and Science (grant CLIOMMICS 01ZX1309); the German Research Council (DFG; Project SI 236/8-1, SI236/9-1, ER 155/6-1, and DFG CRU 216); and the Multiple Myeloma Research Foundation. The German GWAS made use of genotyping data from the population-based HNR study, which is supported by the Heinz Nixdorf Foundation (Germany). The genotyping of the Illumina HumanOmni-1 Quad BeadChips of the HNR subjects was financed by the German Center for Neurodegenerative Disorders (DZNE), Bonn. CoMMpass data were generated as part of the Multiple Myeloma Research Foundation Personalized Medicine Initiatives (https://research.themmrf.org and www.themmrf.org). CIHR funded the Epigenome Mapping Centre at McGill University (grant EP1-120608 awarded to T.P.). We are grateful to all investigators

and patients for their participation. We also thank the clinicians and other staff who contributed to the blood sample and data collection for this study.

Received: May 17, 2017
Revised: July 7, 2017
Accepted: August 18, 2017
Published: September 12, 2017

REFERENCES

Altieri, A., Chen, B., Bermejo, J.L., Castro, F., and Hemminki, K. (2006). Familial risks and temporal incidence trends of multiple myeloma. Eur. J. Cancer *42*, 1661–1670.

Bertolotti, A., Zhang, Y., Hendershot, L.M., Harding, H.P., and Ron, D. (2000). Dynamic interaction of BiP and ER stress transducers in the unfolded-protein response. Nat. Cell Biol. *2*, 326–332.

Broderick, P., Chubb, D., Johnson, D.C., Weinhold, N., Försti, A., Lloyd, A., Olver, B., Ma, Y., Dobbins, S.E., Walker, B.A., et al. (2011). Common variation at 3p22.1 and 7p15.3 influences multiple myeloma risk. Nat. Genet. *44*, 58–61.

Chubb, D., Broderick, P., Frampton, M., Kinnersley, B., Sherborne, A., Penegar, S., Lloyd, A., Ma, Y.P., Dobbins, S.E., and Houlston, R.S. (2015). Genetic diagnosis of high-penetrance susceptibility for colorectal cancer (CRC) is achievable for a high proportion of familial CRC by exome sequencing. J. Clin. Oncol. *33*, 426–432.

Craig, D.W., Liang, W., Venkata, Y., Kurdoglu, A., Aldrich, J., Auclair, D., Allen, K., Harrison, B., Jewell, S., Kidd, P.G., et al. (2013). Interim Analysis Of The Mmrf Commpass Trial, a Longitudinal Study In Multiple Myeloma Relating Clinical Outcomes To Genomic and Immunophenotypic Profiles. Blood *122*, 532–532.

ENCODE Project Consortium (2012). An integrated encyclopedia of DNA elements in the human genome. Nature *489*, 57–74.

Gould, J., Alexanian, R., Goodacre, A., Pathak, S., Hecht, B., and Barlogie, B. (1988). Plasma cell karyotype in multiple myeloma. Blood *71*, 453–456.

Hodson, D.J., Shaffer, A.L., Xiao, W., Wright, G.W., Schmitz, R., Phelan, J.D., Yang, Y., Webster, D.E., Rui, L., Kohlhammer, H., et al. (2016). Regulation of normal B-cell differentiation and malignant B-cell survival by OCT2. Proc. Natl. Acad. Sci. USA *113*, E2039–E2046.

Huang, J., Howie, B., McCarthy, S., Memari, Y., Walter, K., Min, J.L., Danecek, P., Malerba, G., Trabetti, E., Zheng, H.F., et al.; UK10K Consortium (2015). Improved imputation of low-frequency and rare variants using the UK10K haplotype reference panel. Nat. Commun. *6*, 8111.

Kyle, R.A., and Rajkumar, S.V. (2004). Multiple myeloma. N. Engl. J. Med. *351*, 1860–1873.

Kyle, R.A., Remstein, E.D., Therneau, T.M., Dispenzieri, A., Kurtin, P.J., Hodnefield, J.M., Larson, D.R., Plevak, M.F., Jelinek, D.F., Fonseca, R., et al. (2007). Clinical course and prognosis of smoldering (asymptomatic) multiple myeloma. N. Engl. J. Med. *356*, 2582–2590.

Landgren, O., Kristinsson, S.Y., Goldin, L.R., Caporaso, N.E., Blimark, C., Mellqvist, U.H., Wahlin, A., Bjorkholm, M., and Turesson, I. (2009). Risk of plasma cell and lymphoproliferative disorders among 14621 first-degree relatives of 4458 patients with monoclonal gammopathy of undetermined significance in Sweden. Blood *114*, 791–795.

Li, J., Ni, M., Lee, B., Barron, E., Hinton, D.R., and Lee, A.S. (2008). The unfolded protein response regulator GRP78/BiP is required for endoplasmic reticulum integrity and stress-induced autophagy in mammalian cells. Cell Death Differ. *15*, 1460–1471.

Marchini, J., Howie, B., Myers, S., McVean, G., and Donnelly, P. (2007). A new multipoint method for genome-wide association studies by imputation of genotypes. Nat. Genet. *39*, 906–913.

Martincic, K., Alkan, S.A., Cheatle, A., Borghesi, L., and Milcarek, C. (2009). Transcription elongation factor ELL2 directs immunoglobulin secretion in plasma cells by stimulating altered RNA processing. Nat. Immunol. *10*, 1102–1109.

Mifsud, B., Tavares-Cadete, F., Young, A.N., and Sugar, R. (2015). Mapping long-range promoter contacts in human cells with high-resolution capture Hi-C. Nat Genet. *47*, 598–606.

Mitchell, J.S., Li, N., Weinhold, N., Försti, A., Ali, M., van Duin, M., Thorleifsson, G., Johnson, D.C., Chen, B., Halvarsson, B.M., et al. (2016). Genome-wide association study identifies multiple susceptibility loci for multiple myeloma. Nat. Commun. *7*, 12050.

Obeng, E.A., Carlson, L.M., Gutman, D.M., Harrington, W.J., Jr., Lee, K.P., and Boise, L.H. (2006). Proteasome inhibitors induce a terminal unfolded protein response in multiple myeloma cells. Blood *107*, 4907–4916.

Park, K.S., Bayles, I., Szlachta-McGinn, A., Paul, J., Boiko, J., Santos, P., Liu, J., Wang, Z., Borghesi, L., and Milcarek, C. (2014). Transcription elongation factor ELL2 drives Ig secretory-specific mRNA production and the unfolded protein response. J. Immunol. *193*, 4663–4674.

Rao, S.S., Huntley, M.H., Durand, N.C., Stamenova, E.K., Bochkov, I.D., Robinson, J.T., Sanborn, A.L., Machol, I., Omer, A.D., Lander, E.S., and Aiden, E.L. (2014). A 3D map of the human genome at kilobase resolution reveals principles of chromatin looping. Cell *159*, 1665–1680.

Sawyer, J.R., Waldron, J.A., Jagannath, S., and Barlogie, B. (1995). Cytogenetic findings in 200 patients with multiple myeloma. Cancer Genet. Cytogenet. *82*, 41–49.

Scales, M., Chubb, D., Dobbins, S.E., Johnson, D.C., Li, N., Sternberg, M.J., Weinhold, N., Stein, C., Jackson, G., Davies, F.E., et al. (2017). Search for rare protein altering variants influencing susceptibility to multiple myeloma. Oncotarget *8*, 36203–36210.

Smadja, N.V., Fruchart, C., Isnard, F., Louvet, C., Dutel, J.L., Cheron, N., Grange, M.J., Monconduit, M., and Bastard, C. (1998). Chromosomal analysis in multiple myeloma: cytogenetic evidence of two different diseases. Leukemia *12*, 960–969.

Swaminathan, B., Thorleifsson, G., Jöud, M., Ali, M., Johnsson, E., Ajore, R., Sulem, P., Halvarsson, B.M., Eyjolfsson, G., Haraldsdottir, V., et al. (2015). Variants in ELL2 influencing immunoglobulin levels associate with multiple myeloma. Nat. Commun. *6*, 7213.

The 1000 Genomes Project Consortium (2010). A map of human genome variation from population-scale sequencing. Nature *467*, 1061–1073.

Vachon, C.M., Kyle, R.A., Therneau, T.M., Foreman, B.J., Larson, D.R., Colby, C.L., Phelps, T.K., Dispenzieri, A., Kumar, S.K., Katzmann, J.A., and Rajkumar, S.V. (2009). Increased risk of monoclonal gammopathy in first-degree relatives of patients with multiple myeloma or monoclonal gammopathy of undetermined significance. Blood *114*, 785–790.

Vincenz, L., Jäger, R., O'Dwyer, M., and Samali, A. (2013). Endoplasmic reticulum stress and the unfolded protein response: targeting the Achilles heel of multiple myeloma. Mol. Cancer Ther. *12*, 831–843.

Walker, B.A., Wardell, C.P., Murison, A., Boyle, E.M., Begum, D.B., Dahir, N.M., Proszek, P.Z., Melchor, L., Pawlyn, C., Kaiser, M.F., et al. (2015). APOBEC family mutational signatures are associated with poor prognosis translocations in multiple myeloma. Nat Commun. *6*, 6997.

Weinhold, N., Johnson, D.C., Chubb, D., Chen, B., Försti, A., Hosking, F.J., Broderick, P., Ma, Y.P., Dobbins, S.E., Hose, D., et al. (2013). The CCND1 c.870G>A polymorphism is a risk factor for t(11;14)(q13;q32) multiple myeloma. Nat. Genet. *45*, 522–525.

Zhu, Z., Zhang, F., Hu, H., Bakshi, A., Robinson, M.R., Powell, J.E., Montgomery, G.W., Goddard, M.E., Wray, N.R., Visscher, P.M., and Yang, J. (2016). Integration of summary data from GWAS and eQTL studies predicts complex trait gene targets. Nat. Genet. *48*, 481–487.

Cell Reports, Volume *20*

Supplemental Information

Genetic Predisposition to Multiple Myeloma at 5q15 Is Mediated by an *ELL2* Enhancer Polymorphism

Ni Li, David C. Johnson, Niels Weinhold, Scott Kimber, Sara E. Dobbins, Jonathan S. Mitchell, Ben Kinnersley, Amit Sud, Philip J. Law, Giulia Orlando, Matthew Scales, Christopher P. Wardell, Asta Försti, Phuc H. Hoang, Molly Went, Amy Holroyd, Fadi Hariri, Tomi Pastinen, Tobias Meissner, Hartmut Goldschmidt, Kari Hemminki, Gareth J. Morgan, Martin Kaiser, and Richard S. Houlston

Supplemental Information

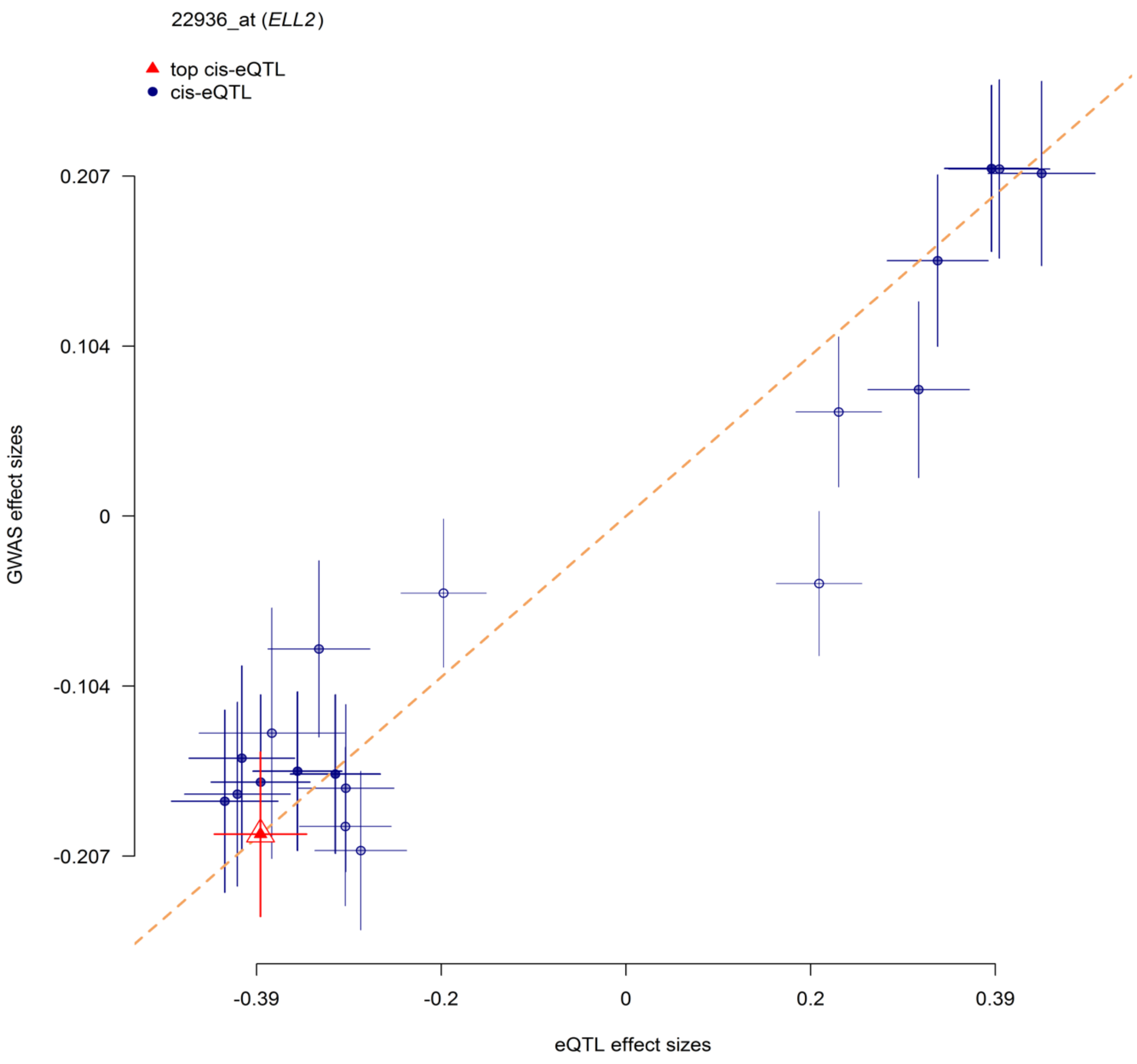

Figure S1, related to Figure 1. Effect sizes of SNPs (used for the HEIDI test) from GWAS meta-analysis plotted against those for SNPs from the respective eQTL study in SMR analysis. The orange dashed lines correspond to the estimate of b_{xy}, at the top *cis*-eQTL. Error bars correspond to standard errors of SNP effects.

Table S1, related to Figure 1. Coverage of sequence variation at 5q15 risk locus by imputation.

Germline variants within chr5:94,700,000-96,000,000 (number of variants (n) = 18,228) were defined by whole-genome sequencing (WGS) data of 640 MM patients sequenced by the CoMMpass Study. To calculate sequence variation coverage by the imputation methods used, biallelic variants from the WGS dataset with minor allele frequency (MAF) > 0.01 and 0.05 were compared with **(A)** the imputation panels (UK10K and 1000 Genome Project) and **(B)** the imputed UK/German GWAS datasets (INFO score > 0.8).

(A) Imputation reference panels (UK10K and 1000 Genome Project)

WGS (Total number of variants called = 18,228)	Coverage of sequence variation
MAF > 0.01, biallelic only (n=4,487)	94.8%
MAF > 0.05, biallelic only (n=2,437)	96.5%

(B) Imputed GWAS datasets (UK or German)

WGS (Total number of variants called = 18,228)	Coverage of sequence variation	
	UK	German
MAF > 0.01, biallelic only (n=4,487)	72.4%	70.3%
MAF > 0.05, biallelic only (n=2,437)	91.1%	90.6%

Table S2, related to Figure 1. Germline coding variants within *ELL2*.

Shown is the association of coding variants within *ELL2* with MM risk, assessed with 513 MM cases from the UK MyIX and MyXI clinical trials and 1,569 UK controls from the UK 1958 Birth Cohort (Scales et al., 2017). Position of SNP based on NCBI build 37 of the human genome. r^2 with respect to rs11372862. REF, reference allele; ALT, alternate allele with their allele counts in case and control populations. Also shown the MAF; minor allele frequency for cases and controls. *P*-value derived from Fisher's exact test (two-sided) assessing the difference in allele frequency in cases and controls. NA, SNP ID not available. Asterisk (*) denotes variants with no call rate of > 0.03 across either case or control samples.

					Cases			Controls			
**Variant**	**Position (bp)**	r^2	**REF**	**ALT**	**REF**	**ALT**	**MAF**	**REF**	**ALT**	**MAF**	***P*-value**
rs141910684	95234139	<0.001	G	A	819*	1*	0.0012	3086	2	0.00065	0.51
rs3777204	95234350	0.91	A	G	772	230	0.23	2187	849	0.28	1.76×10^{-3}
rs3777203	95234377	0.92	C	T	785	219	0.22	2110*	822*	0.28	9.71×10^{-5}
rs3777202	95234392	0.92	A	C	795	223	0.22	1953*	815*	0.29	3.34×10^{-6}
rs17085249/COSM1132083	95236415	0.92	G	A	788	210	0.21	2308	828	0.26	6.80×10^{-4}
rs41276265/COSM1620674	95236454	0.06	G	C	997	13	0.013	3080	56	0.018	0.32
rs3815768	95236459	0.92	C	T	791	213	0.21	2307	827	0.26	9.54×10^{-4}
rs113894818	95236474	0.007	G	T	986	14	0.014	3063	61	0.020	0.28
NA	95242281	<0.001	C	G	1021	1	0.00098	2228*	0*	0	0.31
NA	95242383	<0.001	T	C	1021	1	0.00098	3066	0	0	0.25
rs139040465	95249572	0.003	G	A	1022	2	0.0020	3119	7	0.0022	1.00
NA	95249586	<0.001	C	T	1025	1	0.00097	3090	0	0	0.25
rs146249322	95278729	0.02	T	C	990	4	0.0040	3111	23	0.0073	0.37

Table S3, related to Figure 1. Subtype case-control and case-only analyses at 5q15.

(A) Subtype case-control analysis for MM hyperdiploid (HRDMM) (1,363 cases, 7,304 controls) and for non-HRDMM cases (1,339 cases, 7,304 controls).

(B) Subtype case-only analysis for 1,363 HRDMM cases.

Position of SNP based on NCBI build 37 of the human genome. Odds ratio (OR) derived relative to the risk allele with 95% confidence interval (CI) shown. Meta *P*-value derived from meta-analysis of UK and German GWAS datasets. P_{het} represents Cochran's test of between-study heterogeneity *P*-value. I^2 denotes the proportion of the total variation due to heterogeneity, with values ≥75% considered to be characteristic of large heterogeneity.

(A) Case-control

SNP	Position (bp)	Risk allele	OR	95% CI	Meta *P*-value	P_{het}	I^2
**HRDMM**							
rs1423269	95255724	A	1.24	1.12-1.36	2.85×10^{-5}	0.86	0%
rs11372862	95257775	A	1.26	1.14-1.41	1.37×10^{-5}	0.52	0%
**Non-HRDMM**							
rs1423269	95255724	A	1.09	0.98-1.20	0.11	0.64	0%
rs11372862	95257775	A	1.12	1.00-1.25	0.05	0.88	0%

(B) Case-only

SNP	Position (bp)	Risk allele	OR	95% CI	Meta *P*-value	P_{het}	I^2
rs1423269	95255724	A	1.11	1.00-1.25	0.06	0.61	0%
rs11372862	95257775	A	1.13	1.00-1.28	0.04	0.37	0%

Table S4, related to Figure 2. SNPs within *ELL2* enhancer correlated with rs11372862 (r^2≥0.8).

Position of SNP based on NCBI build 37 of the human genome. r^2 with respect to rs11372862. Odds ratio (OR) derived relative to the risk allele with 95% confidence interval (CI) shown. Meta *P*-value derived from **(A)** meta-analysis of the UK MyIX and MyXI and German GMMG GWAS with 3,790 cases and 7,304 controls, **(B)** meta-analysis of HRDMM case-control analyses from the UK and German populations with 1,363 cases and 7,304 controls.

Emboldened are SNPs within interacting fragment with *ELL2* promoter (chr5:95,260,175-95,264,576; * in **Figure 2**). P_{het} represents Cochran's test of between-study heterogeneity *P*-value. I^2 denotes the proportion of the total variation due to heterogeneity, with values ≥75% considered to be characteristic of large heterogeneity.

(A) UK and German GWAS

SNP	Position (bp)	r^2	Risk allele	OR	95% CI	Meta *P*-value	P_{het}	I^2
rs3777185	95259862	0.97	C	1.15	1.07-1.22	6.11×10^{-5}	0.87	0%
**rs6877329**	**95261538**	**0.93**	**C**	**1.14**	**1.07-1.22**	$\mathbf{8.24\times10^{-5}}$	**0.62**	**0%**
**rs3777184**	**95262044**	**0.97**	**G**	**1.15**	**1.07-1.23**	$\mathbf{5.91\times10^{-5}}$	**0.88**	**0%**
rs889302	95264652	0.92	A	1.14	1.07-1.22	7.44×10^{-5}	0.65	0%
rs2015159	95265087	0.92	C	1.14	1.07-1.22	7.21×10^{-5}	0.65	0%
rs4563648	95265556	0.92	G	1.14	1.07-1.22	6.42×10^{-5}	0.65	0%

(B) UK and German HRDMM case-control

SNP	Position (bp)	r^2	Risk allele	OR	95% CI	Meta *P*-value	P_{het}	I^2
rs3777185	95259862	0.97	C	1.24	1.12-1.36	2.75×10^{-5}	0.88	0%
**rs6877329**	**95261538**	**0.93**	**C**	**1.22**	**1.10-1.34**	$\mathbf{9.65\times10^{-5}}$	**0.51**	**0%**
**rs3777184**	**95262044**	**0.97**	**G**	**1.24**	**1.12-1.37**	$\mathbf{2.38\times10^{-5}}$	**0.88**	**0%**
rs889302	95264652	0.92	A	1.22	1.10-1.34	8.73×10^{-5}	0.61	0%
rs2015159	95265087	0.92	C	1.22	1.10-1.34	8.16×10^{-5}	0.61	0%
rs4563648	95265556	0.92	G	1.22	1.11-1.35	7.00×10^{-5}	0.60	0%

Table S5, related to Figure 3 and 4. eQTL analyses.

(A) The relationship between rs1423269, rs11372862, rs6877329 genotypes and *ELL2* expression was assessed in hyperdiploid (HRDMM) and non-HRDMM CD138-selected MM plasma cells from 170 UK cases (GEO: GSE21349) and 602 German MM patients (EMBL-EBI: E-MTAB-2299). Association between SNP genotype and *ELL2* expression (22936_at) was evaluated based on the significance of linear regression coefficients. Meta *P*-value derived from meta-analysis of the UK and German datasets. Direction of eQTL is shown with respect to the risk alleles.

(B) The relationship between rs1423269, rs11372862, rs6877329 genotypes and genes with which *ELL2* is correlated in expression (*BiP*, *ATF6* and *POU2AF1* in **Figure 4**) was assessed in hyperdiploid (HRDMM) and non-HRDMM CD138-selected MM plasma cells from 170 UK cases (GEO: GSE21349) and 602 German MM patients (EMBL-EBI: E-MTAB-2299). *ELL1* expression data is not available as not expressed in MM patients ($\log_2$ expression < 3.5 in ≥ 95% samples). Association between SNP genotype and gene expression was evaluated based on the significance of linear regression coefficients. Meta *P*-value derived from meta-analysis of the UK and German datasets.

(A)

SNP	Position (bp)	Risk allele	Meta *P*-value	Direction
**HRDMM**				
rs1423269	95255724	A	4.45×10^{-10}	Decrease
rs11372862	95257775	A	1.40×10^{-10}	Decrease
rs6877329	95261538	C	1.35×10^{-9}	Decrease
**Non-HRDMM**				
rs1423269	95255724	A	1.60×10^{-6}	Decrease
rs11372862	95257775	A	1.53×10^{-6}	Decrease
rs6877329	95261538	C	4.07×10^{-7}	Decrease

(B)

SNP	Position (bp)	Risk allele	Meta *P*-value		
			BiP (3309_at)	*ATF6* (22926_at)	*POU2AF1* (5450_at)
**HRDMM**					
rs1423269	95255724	A	0.17	0.95	0.39
rs11372862	95257775	A	0.78	0.72	0.57
rs6877329	95261538	C	0.28	1.00	0.48
**Non-HRDMM**					
rs1423269	95255724	A	0.65	0.38	0.63
rs11372862	95257775	A	0.94	0.18	0.69
rs6877329	95261538	C	0.60	0.57	0.55

Table S6, related to Figure 3. Investigating preferential amplification of the risk allele.

Number of HRDMM samples, heterozygous for the risk allele, with somatic amplification of the risk (AAB). *P*-value derived from exact binomial test of consensus.

	Inferred state		
	AAB	ABB	*P*-value
**MyXI Exomes**	34	23	0.18
**CoMMpass Exomes**	27	23	0.67

Table S7, related to Figure 4. Effect of rs6877329 genotypes and *ELL2*, *BiP*, *ATF6*, *ELL1* and *POU2AF1* expression on patient survival.

(A) Data from the UK-GWAS (1,165 cases from the UK MRC Myeloma IX trial, UK-MyIX; 877 cases from the UK MRC Myeloma XI trial, UK-MyXI) and the German-GWAS (511 cases from the GER-GMMG trial). Shown is the hazard ratio (HR) relative to the C-risk allele with associated 95% confidence intervals (CI).

Overall survival and progression-free survival analyses comparing patients with upper and lower quartile of **(B)** *ELL2*, **(C)** *BiP*, **(D)** *ATF6* and **(E)** *ELL1* and **(F)** *POU2AF1* expression. Data from 259 MM individuals from the UK MRC Myeloma IX trial (UK-MyIX; GEO: GSE21349) and 246 MM individuals from the German GMMG clinical trial (GER-GMMG; EMBL-EBI: E-MTAB-372) with expression data. Shown is the hazard ratio (HR) with associated 95% confidence intervals (CI).

(A) Relationship between rs6877329 genotypes and patient survival

Datasets		Overall survival	Progression-free survival
UK-MyIX	HR (95% CI)	0.95 (0.83-1.09)	0.98 (0.89-1.10)
	P-value	0.47	0.80
UK-MyXI	HR (95% CI)	1.00 (0.80-1.26)	1.03 (0.88-1.20)
	P-value	0.99	0.71
GER-GMMG	HR (95% CI)	0.96 (0.74-1.26)	0.96 (0.81-1.15)
	P-value	0.80	0.66

(B) Relationship between *ELL2* expression and patient survival

Datasets		Overall survival	Progression-free survival
UK-MyIX	HR (95% CI)	0.90 (0.59-1.37)	1.16 (0.81-1.67)
	P-value	0.62	0.41
GER-GMMG	HR (95% CI)	1.06 (0.55-2.07)	0.89 (0.56-1.42)
	P-value	0.85	0.63

(C) Relationship between *BiP* expression and patient survival

Datasets		Overall survival	Progression-free survival
UK-MyIX	HR (95% CI)	0.78 (0.52-1.17)	0.86 (0.60-1.23)
	P-value	0.23	0.41
GER-GMMG	HR (95% CI)	0.75 (0.40-1.42)	0.77 (0.49-1.21)
	P-value	0.38	0.25

(D) Relationship between *ATF6* expression and patient survival

Datasets		Overall survival	Progression-free survival
UK-MyIX	HR (95% CI)	0.89 (0.60-1.34)	0.84 (0.59-1.21)
	P-value	0.59	0.35
GER-GMMG	HR (95% CI)	1.09 (0.58-2.07)	1.02 (0.64-1.61)
	P-value	0.79	0.94

(E) Relationship between *ELL1* expression and patient survival

Datasets		Overall survival	Progression-free survival
UK-MyIX	HR (95% CI)	0.99 (0.66-1.48)	0.93 (0.65-1.32)
	P-value	0.96	0.68
GER-GMMG	HR (95% CI)	1.10 (0.57-2.15)	0.95 (0.59-1.53)
	P-value	0.77	0.82

(F) Relationship between *POU2AF1* expression and patient survival

Datasets		Overall survival	Progression-free survival
UK-MyIX	HR (95% CI)	1.14 (0.76-1.72)	1.25 (0.87-1.80)
	P-value	0.52	0.23
GER-GMMG	HR (95% CI)	1.02 (0.55-1.90)	1.25 (0.80-1.96)
	P-value	0.95	0.33

Supplemental Experimental Procedures

Ethics

Collection of patient samples and associated clinico-pathological information was undertaken with written informed consent and relevant ethical review board approval at respective study centers in accordance with the tenets of the Declaration of Helsinki. Specifically, the MRC Leukemia Data Monitoring and Ethics committee (MREC 02/8/95, ISRCTN68454111, MREC 17/09/09, ISRCTN49407852) and University of Heidelberg Ethical Commission (229/2003, S-337/2009, AFmu-119/2010).

Genome-wide association study data

The UK-GWAS and German-GWAS of MM have been previously reported (Mitchell et al., 2016). Briefly, the UK-GWAS comprised 2,329 MM cases, including 702 with HRDMM, genotyped using Illumina Human OmniExpress-12 v1.0 arrays (Illumina, San Diego). Cases were recruited through the UK MRC Myeloma-IX (MyIX) and Myeloma-XI (MyXI) trials (ISRCTN68454111: MyIX http://www.isrctn.com/search?q=ISRCTN68454111 and ISRCTN49407852: MyXI http://www.isrctn.com/search?q=ISRCTN49407852). Controls were provided by the Wellcome Trust Case Control Consortium 2 (http://www.wtccc.org.uk/) with 2,698 individuals in the 1958 British Birth Cohort genotyped using Hap1.2M-Duo Custom array data and 2,501 individuals from the UK Blood Service.

The German-GWAS comprised 1,512 MM cases, including 661 with HRDMM, genotyped using Illumina Human OmniExpress-12v1.0 arrays. Cases were recruited by the GMMG coordinated by the University Clinic, Heidelberg (ISRCTN06413384: GMMG-HD3 http://www.isrctn.com/search?q=ISRCTN06413384; ISRCTN64455289: GMMG-HD4 http://www.isrctn.com/search?q=ISRCTN64455289; and ISRCTN05745813: GMMG-HD5 http://www.isrctn.com/search?q=ISRCTN05745813). Controls comprised 2,107 healthy individuals from the Heinz Nixdorf Recall study genotyped using Illumina HumanOmni1-Quadv1 and HumanOmniExpress-12v1.0 arrays.

GWAS quality control has been described previously (Mitchell et al., 2016). To recover untyped genotypes we performed imputation using IMPUTE2 v2.3 with a combined UK10K and 1000 Genomes Project (phase 1 integrated release 3, March 2012) panel for reference (1000Genomes, 2010; Huang et al., 2015). Poorly imputed SNPs (INFO score < 0.80) were excluded. To assess sequence variation coverage by the GWAS arrays and current imputation methods we used WGS data from 640 MM patients from the CoMMpass Study (Craig et al., 2013). Genotypes of 18,228 variants called from the WGS data within chr5:94,700,000-96,000,000 were compared with the imputation reference panels used and the imputed genotypes from UK and German GWAS datasets.

Frequentist association testing between SNP genotype and MM was performed using logistic regression under an additive genetic model in SNPTESTv2.5 (Marchini et al., 2007). Meta-analysis was undertaken under a fixed-effects model using inverse variance weighting in META v1.7 (Marchini et al., 2007). To look for independent effects, conditional analysis was performed with SNPTESTv2.5 with genotypes from UK and German GWAS individuals conditioning on rs11376892. Association of germline coding variants within *ELL2* with MM risk was estimated using germline WES data from 513 MM patients and 1,569 controls as detailed in Scales et al., 2017. Fisher's exact test (two-sided) was used to assess the difference in allele frequency of the coding variants in cases and controls.

Subtype detection

Karotyping was used for cytogenetic studies of MM cells and standard criteria for the definition of a clone were applied. Fluorescence *in situ* hybridization (FISH) and ploidy classification of UK samples was conducted using the methodologies previously described (Chiecchio et al., 2006). FISH and ploidy classification of German samples was performed as previously described (Neben et al., 2010). Logistic regression in case-only and case-control analyses was used to assess tumor karyotype. Meta-analysis was undertaken under a fixed-effects model using inverse variance weighting in META v1.7 (Marchini et al., 2007).

Expression quantitative trait loci analysis

eQTL analyses were performed for CD138-purified plasma cells from 183 MRC MyIX trial patients (GEO: GSE21349) and 658 Heidelberg GMMG patients (EMBL-EBI: E-MTAB-2299) using Affymetrix Human Genome U133 2.0 Plus Array (Weinhold et al., 2015). Briefly, German and UK data were pre-processed separately, followed by analysis using a Bayesian approach to probabilistic estimation of expression residuals to infer broad variance components, accounting for hidden determinants influencing global expression. The association between genotype of SNPs and expression of genes within 500 kb either side of rs11372862 was evaluated based on the significance of linear regression coefficients. We pooled data from the two studies under a fixed-effects model. For subtype-specific eQTL, association analyses were performed between *ELL2* (22936_at), *BiP* (3309_at), *ATF6* (22926_at) and *POU2AF1* (5450_at) expression and SNPs rs11372862, rs1423269 or rs6877329 for 170 MRC MyIX trial patients and 602 Heidelberg GMMG patients with karyotype and expression data.

We carried out Summary data-based Mendelian randomization (SMR) analysis using previously established methods (Zhu et al., 2016). As previously advocated only probes with at least one eQTL *P*-value of $< 5.0 \times 10^{-8}$ were considered for SMR analysis. We set a threshold for the SMR test of $pSMR < 0.00833$ corresponding to a Bonferroni correction for 6 tests (6 probesets within 1 MB of rs11372862). We generated plots of the eQTL and GWAS associations at the locus, as well as plots of GWAS and eQTL effect sizes (i.e. corresponding to input for the HEIDI heterogeneity test). HEIDI test *P*-values < 0.05 were taken to indicate significant heterogeneity.

Cell lines

Human MM cell line KMS11 was obtained from the American Type Culture Collection (ATCC) and grown in Advanced RPMI 1640 Medium supplemented with GlutaMAX and 10% heat-inactivated fetal bovine serum (FBS). Human PCL cell lines L363 and JJN3 were obtained from Deutsche Sammlung von Mikroorganism und Zellkulturen GmbH (DSMZ). L363 was grown in Advanced RPMI 1640 supplemented with GlutaMAX and 15% heat-inactivated FBS. JJN3 was grown in 40% Dulbecco's MEM, 40% Iscove's MDM supplemented with 20% heat-inactivated FBS. Human lymphoblastoid cell line GM11992 were obtained from Coriell Institute and grown in RPMI 1640 culture medium supplemented with 15% FBS. Cell culture media were obtained from Life Technologies (Carlsbad, CA, USA) and FBS from PAA Laboratories (Pasching, Austria). Cells were cultured at 37°C, 100% relative humidity and 5% CO_2. Cell lines used were confirmed to be mycoplasma-free (PCR Mycoplasma Test Kit I/C, PromoCell, Heidelberg, Germany). Genotype of rs6877329 and rs3777184 in KMS11 and GM11992 was confirmed by Sanger sequencing using sequencing primers detailed below.

**Sequencing primers**	
rs3777184 F	GGAGTAGGAAGGGGAGGGTA
rs3777184 R	ATTGGCAGTTATCCCCAAAA
rs6877329 F	GAGGGGTTCTTTCCTCAAAT
rs6877329 R	CAAACTTACTGCAAACATTTAAACA

ENCODE and chromatin state dynamics

To explore the epigenetic profile of association signals at 5q15, we used DNAseI HS, transcription factor ChIP-seq data, histone modifications (H3K4Me1, H3K4Me3 and H3K27Ac) and ChromHMM in GM12878 from the ENCODE project (ENCODE, 2012). ChIP-seq on H3K4Me1, H3K4Me3 and H3K27Ac histone modifications were carried out in MM cell line KMS11 and PCL cell lines L363 and JJN3. Antibodies were obtained from Diagenode (Seraing, Belgium) for H3K4Me1 (C15410194), H3K4Me3 (C15410003) and H3K27Ac (C15410196) histone modifications. We used the *in silico* algorithms SIFT (Kumar et al., 2009) and PolyPhen-2 (Adzhubei et al., 2010) to predict the impact of amino acid substitutions.

Briefly, after cell lysing, sonication of nuclei was performed (BioRuptor; Diagenode, Liege, Belgium) to obtain 150-500 bp fragments. ChIP reactions were performed on a Diagenode SX-8G IP-Star Compact using Diagenode automated Ideal Kit reagents (C01010011, Diagenode). Protein A beads were incubated for 10 hours with 3-6 μg of

antibody and 2-4 million of sonicated cell lysate. ChIP samples were de-crosslinked at 65°C for 4 hours and subsequently treated for 30 minutes with RNAse Cocktail and Proteinase K. DNA was then purified (MiniElute PCR purification kit, Qiagen, Hilden, Germany), followed by library preparation according to manufacture (HTP Illumina library preparation kit, KAPA biosystem; Massachusetts, USA). Fourteen cycles of PCR were performed, followed by size selection for 200-400 bp fragments and final library purification (GeneRead Size Selection kit, Qiagen). ChIP libraries were sequenced using Illumina HiSeq 2000 (Illumina) with 100 bp single-ended reads. Generated raw reads were filtered for quality (Phred33 $\geq$ 30) and length (n $\geq$ 32), and adapter sequences were removed using Trimmomatic v0.22 (Lohse et al., 2012). Reads passing filters were then aligned to the human reference (hg19) using BWA v0.6.1. Peak calls are obtained using MACS2 v 2.0.10.07132012 (Feng et al., 2012) .

In situ promoter capture Hi-C

In situ promoter capture Hi-C was conducted in the cell line KMS11. *In situ* Hi-C libraries were prepared as previously described (Rao et al., 2014). To increase cell lysis efficiency, 3 aliquots of 8 million cells were fixed separately in 1% formaldehyde for 10 mins, each aliquot lysed in 15 mL lysis buffer and incubated on ice for 1 hour before being combined. Cross-linked DNA was digested by restriction enzyme *Hind*III (NEB, Ipswich, USA). Digested chromatin ends were filled and marked with biotin-14-dATP (ThermoFisher Scientific, Waltham, USA). The resulting blunt end fragments were ligated at 16°C in the nucleus with T4 DNA ligase (NEB) to minimize random ligation. DNA purified after crosslinking was reversed by proteinase K (Ambion, ThermoFisher Scientific) treatment. DNA was sheared by sonication (Covaris, Massachusetts, USA) and approximately 200-650 bp fragments selected. Biotin tagged DNA was pulled down with streptavidin beads and ligated with Illumina paired end adapters. Six cycles of PCR were performed to amplify libraries before capture. Promoter capture was based on 32,313 biotinylated 120-mer RNA baits (Agilent, Santa Clara, USA) targeting both ends of *Hind*III restriction fragments that overlap Ensembl promoters of protein-coding, non-coding, antisense, snRNA, miRNA and snoRNA transcripts. After library enrichment, a post-capture PCR step was carried out using 5 amplification cycles. Hi-C and CHi-C libraries were sequenced using Illumina HiSeq 2000 technology. Reads were aligned to the GRCh37 build using bowtie2 v2.2.6 and identification of valid di-tags was performed using HICUP v0.5.9. To declare significant contacts, HiCUP output was processed using CHiCAGO v1.1.8. Data from three independent biological replicates were combined to obtain a definitive set of contacts. The interaction within *ELL2* from its promoter with the highest score was plotted. Promoter capture Hi-C on GM12878 was obtained from publically available EMBL-EBI: E-MTAB-2323 (Mifsud et al., 2015).

Plasmid construction and luciferase assays

A 1 kb regulatory region with the risk alleles of rs6877329 and rs3777184 was amplified from human genomic DNA using primers detailed below, cloned into the PCR8/GW/TOPO vector and then transferred into pGL3 *luc*+ SV40-promoter vector using Gateway technology (Life Technologies). The non-risk alleles of rs6877329 and rs3777184 were generated with site-directed mutagenesis using Quick Change XL (Agilent, Santa Clara, USA). Reporter constructs were introduced into KMS11 by nucleofection, using program X-01 on the Amaxa Nucleofector I (Amaxa Biosystems, Lonza, Basel, Switzerland). 5×10^6 cells were resuspended in 100 µl Cell Line Nucleofector Solution V and mixed with 3 µg reporter plasmid DNA and 60 ng of internal control plasmid (pRL-SV40). Transiently transfected cells were grown for 24 hours before assaying with the Dual-Luciferase Reporter Assay System (Promega, Madison, USA) and the Fluoroskan Ascent FL plate reader (ThermoFisher Scientific). Relative luciferase activity was calculated as the ratio of luminescence from the experimental reporter to the internal control plasmid (pRL-SV40). Statistical significance was calculated using the two-tailed *t*-test over three biological replicates.

Plasmid construction primers	
ELL2 amplicon F	TAACTTTCTTTGCTGTGGGT
ELL2 amplicon R	GAATTTTGGGGATAACTGC
SDM rs6877329 F	CGGCATCCCAGGAAGGCAGTCCATCTCTTAA
SDM rs6877329 R	TTAAGAGATGGACTGCCTTCCTGGGATGCCG
SDM rs3777184 F	GGAAGGGCAGAGGGTACAGAGGATCCAACAC
SDM rs3777184 R	GTGTTGGATCCTCTGTACCCTCTGCCCTTCC
Sequencing F	TAACTTTCTTTGCTGTGGGT
Sequencing R	GAATTTTGGGGATAACTGC

Electrophoretic mobility shift assay

Nuclear protein was extracted from GM11992 cells using NE-PER nuclear and cytoplasmic extraction kit (ThermoFisher Scientific). Infrared dye DY-682-labeled (Eurofins Genomics, Germany) and unlabeled (Life Technologies) complementary oligonucleotides flanking rs6877329 were annealed to generate double-stranded EMSA probes detailed below. Each 20 µl binding reaction contained 50 fmol labeled target DNA, 1 × binding buffer (10 mM Tris, 50 mM KCl, 1 mM DTT [pH 7.5], 1 µg poly [dI.dC, Sigma-Aldrich], 2.5 mM DTT, and 10 µg nuclear protein extract). The reaction mix was incubated in the dark for 30 minutes at room temperature. Competition assays were performed by adding 100-fold molar excess of unlabeled probes. Super-shifts were performed by adding 2 µg CEBPβ antibody (sc-376591; Santa Cruz Biotechnology, Texas, USA) to the binding reaction and incubating for 15 minutes before the addition of labeled probe. DNA-protein complexes were resolved by electrophoresis on a 6% DNA retardation gel (Life Technologies) in 1× Tris-borate-EDTA (TBE) at 4 °C. Gels were imaged using the Odyssey Fc Infrared Imaging System (LI-COR Biosciences, Nebraska, USA).

EMSA oligos	
rs6877329 C F	CCCTCCGGCATCCCAGGAAGCCAGTCCATCTCTTAATTTTA
rs6877329 C R	TAAAATTAAGAGATGGACTGGCTTCCTGGGATGCCGGAGGG
rs6877329 G F	CCCTCCGGCATCCCAGGAAGGCAGTCCATCTCTTAATTTTA
rs6877329 G R	TAAAATTAAGAGATGGACTGCCTTCCTGGGATGCCGGAGGG

Gene expression analyses

We assessed the relationship between *ELL2* and *BiP*, *ATF6*, *POU2AF1*, *ELL1* gene expression (log_2-transformed) in two independent MM data sets GEO: GSE21349 (259 patients from the UK MRC MyIX clinical trial) and EMBL-EBI: E-MTAB-372 (246 patients from the German GMMG clinical trial) using Pearson's product-moment correlation test. Expression data generated using Affymetrix Human Genome U133 2.0 Plus Array. *P*-values from the two patient data sets were combined by Fisher's Method. Statistical tests were conducted using the R software version 3.1.3.

Relationship between SNP genotype and somatic copy number

To investigate whether the risk SNP rs6877329 was preferentially amplified in heterozygous individuals with trisomy of chromosome 5 we analyzed previously published WES data on 463 MyXI trial cases (Walker et al., 2015) in conjunction with WGS and WES data on 664 cases produced by the CoMMpass Study (Craig et al., 2013). Hyperdiploid cases were identified with chromosome 5 trisomy and amplification of at least one other odd numbered chromosome.

MyXI trial data: ExomeCNV was used to define amplification (logR > 0.1) (Sathirapongsasuti et al., 2011). Samples were identified as heterozygous for the risk SNP if heterozygous for five coding SNPs in LD with the risk variant (r^2 > 0.95) (rs17085249, rs3815768, rs3777202, rs3777203, rs3777204).

CoMMpass data: Amplification was defined using CBS (Venkatraman and Olshen, 2007) designated copy number segments called by CoMMpass (> 50% chromosome segment.mean > 0.3). WGS and WES reads were aligned to

GRCh37 build using BWA software (Li et al., 2009) and variants called according to GATK best practices (DePristo et al., 2011). As the whole genome sequencing data was low coverage, the germline genotype of the risk variant was determined using itself and an additional 18 SNPs in LD ($r^2 > 0.99$).

57 and 50 hyperdiploid samples heterozygous for the risk variant with chromosome 5 trisomy were identified in the MyXI and CoMMpass data sets respectively. Bam-readcounts (https://github.com/genome/bam-readcount/) was used to extract the counts of reads supporting the risk and non-risk variant from the WES data at 2/5 of the coding SNPs aforementioned, separated sufficiently to out rule common reads (rs3777204 and rs3815768, separated by > 1 kb). Given the counts of risk and non-risk reads sampled at these positions, each sample was assigned to its most probable state (amplification of the risk or non-risk allele) assuming a binomial distribution of counts. In germline heterozygote samples a bias towards the mapping of a reference read was observed. An adjustment to the probabilities was therefore made using the germline ratio of reference to alternate reads to estimate the mapping bias for each SNP.

Association between rs6877329 genotypes, *ELL2* expression and patient outcome

To examine the relationship between *ELL2*, *BiP*, *ATF6*, *ELL1* and *POU2AF1* expression and patient outcome, we used data from two independent patient cohorts (GEO: GSE21349, 259 patients from the UK MRC MyIX clinical trial; EMBL-EBI: E-MTAB-372, 246 patients from the German GMMG clinical trial). For each data set, patients were grouped by *ELL2*, *BiP*, *ATF6*, *ELL1* or *POU2AF1* expression (upper and lower quartiles). Analysis was performed using the log-rank test to estimate expression-associated hazard ratio and 95% confidence intervals. To examine the relationship between rs6877329 genotype and patient outcome, we analyzed GWAS data on the three patient cohorts specifically (i) 1,165 cases in the UK GWAS from the UK MRC MyIX trial; (ii) 877 MM cases in the UK GWAS from the UK MRC MyXI trial; (iii) 511 of the patients in the German GWAS from the German GMMG clinical trial. Analysis end points were myeloma-specific overall survival and progression-free survival, and analysis was performed as previously described (Johnson et al., 2016). Cox regression analysis was used to derive genotype-specific hazard ratio and associated 95% confidence intervals.

Supplemental References

1000Genomes (2010). A map of human genome variation from population-scale sequencing. Nature *467*, 1061-1073.

Adzhubei, I.A., Schmidt, S., Peshkin, L., Ramensky, V.E., Gerasimova, A., Bork, P., Kondrashov, A.S., and Sunyaev, S.R. (2010). A method and server for predicting damaging missense mutations. Nature methods *7*, 248-249.

Chiecchio, L., Protheroe, R.K., Ibrahim, A.H., Cheung, K.L., Rudduck, C., Dagrada, G.P., Cabanas, E.D., Parker, T., Nightingale, M., Wechalekar, A., *et al.* (2006). Deletion of chromosome 13 detected by conventional cytogenetics is a critical prognostic factor in myeloma. Leukemia *20*, 1610-1617.

Craig, D.W., Liang, W., Venkata, Y., Kurdoglu, A., Aldrich, J., Auclair, D., Allen, K., Harrison, B., Jewell, S., Kidd, P.G., *et al.* (2013). Interim Analysis Of The Mmrf Commpass Trial, a Longitudinal Study In Multiple Myeloma Relating Clinical Outcomes To Genomic and Immunophenotypic Profiles. Blood *122*, 532-532.

DePristo, M.A., Banks, E., Poplin, R., Garimella, K.V., Maguire, J.R., Hartl, C., Philippakis, A.A., del Angel, G., Rivas, M.A., Hanna, M., *et al.* (2011). A framework for variation discovery and genotyping using next-generation DNA sequencing data. Nature genetics *43*, 491-+.

ENCODE (2012). An integrated encyclopedia of DNA elements in the human genome. Nature *489*, 57-74.

Feng, J., Liu, T., Qin, B., Zhang, Y., and Liu, X.S. (2012). Identifying ChIP-seq enrichment using MACS. Nature protocols *7*, 1728-1740.

Huang, J., Howie, B., McCarthy, S., Memari, Y., Walter, K., Min, J.L., Danecek, P., Malerba, G., Trabetti, E., Zheng, H.F., *et al.* (2015). Improved imputation of low-frequency and rare variants using the UK10K haplotype reference panel. Nat Commun *6*, 8111.

Johnson, D.C., Weinhold, N., Mitchell, J.S., Chen, B., Kaiser, M., Begum, D.B., Hillengass, J., Bertsch, U., Gregory, W.A., and Cairns, D. (2016). Genome-wide association study identifies variation at 6q25.1 associated with survival in multiple myeloma. Nat Comm *7*, 10290.

Kumar, P., Henikoff, S., and Ng, P.C. (2009). Predicting the effects of coding non-synonymous variants on protein function using the SIFT algorithm. Nature protocols *4*, 1073-1081.

Li, H., Handsaker, B., Wysoker, A., Fennell, T., Ruan, J., Homer, N., Marth, G., Abecasis, G., Durbin, R., and Genome Project Data Processing, S. (2009). The Sequence Alignment/Map format and SAMtools. Bioinformatics *25*, 2078-2079.

Lohse, M., Bolger, A.M., Nagel, A., Fernie, A.R., Lunn, J.E., Stitt, M., and Usadel, B. (2012). RobiNA: a user-friendly, integrated software solution for RNA-Seq-based transcriptomics. Nucleic acids research *40*, W622-627.

Marchini, J., Howie, B., Myers, S., McVean, G., and Donnelly, P. (2007). A new multipoint method for genome-wide association studies by imputation of genotypes. Nature genetics *39*, 906-913.

Mifsud, B., Tavares-Cadete, F., Young, A.N., and Sugar, R. (2015). Mapping long-range promoter contacts in human cells with high-resolution capture Hi-C. *47*, 598-606.

Mitchell, J.S., Li, N., Weinhold, N., Forsti, A., Ali, M., van Duin, M., Thorleifsson, G., Johnson, D.C., Chen, B., Halvarsson, B.M., *et al.* (2016). Genome-wide association study identifies multiple susceptibility loci for multiple myeloma. Nat Commun *7*, 12050.

Neben, K., Jauch, A., Bertsch, U., Heiss, C., Hielscher, T., Seckinger, A., Mors, T., Muller, N.Z., Hillengass, J., Raab, M.S., *et al.* (2010). Combining information regarding chromosomal aberrations t(4;14) and del(17p13) with the International Staging System classification allows stratification of myeloma patients undergoing autologous stem cell transplantation. Haematologica *95*, 1150-1157.

Rao, S.S., Huntley, M.H., Durand, N.C., Stamenova, E.K., Bochkov, I.D., Robinson, J.T., Sanborn, A.L., Machol, I., Omer, A.D., Lander, E.S., *et al.* (2014). A 3D map of the human genome at kilobase resolution reveals principles of chromatin looping. Cell *159*, 1665-1680.

Sathirapongsasuti, J.F., Lee, H., Horst, B.A., Brunner, G., Cochran, A.J., Binder, S., Quackenbush, J., and Nelson, S.F. (2011). Exome sequencing-based copy-number variation and loss of heterozygosity detection: ExomeCNV. Bioinformatics *27*, 2648-2654.

Scales, M., Chubb, D., Dobbins, S.E., Johnson, D.C., Li, N., Sternberg, M.J., Weinhold, N., Stein, C., Jackson, G., Davies, F.E., *et al.* (2017). Search for rare protein altering variants influencing susceptibility to multiple myeloma. Oncotarget *8*, 36203-36210.

Venkatraman, E.S., and Olshen, A.B. (2007). A faster circular binary segmentation algorithm for the analysis of array CGH data. Bioinformatics *23*, 657-663.

Walker, B.A., Wardell, C.P., Murison, A., Boyle, E.M., Begum, D.B., Dahir, N.M., Proszek, P.Z., Melchor, L., Pawlyn, C., Kaiser, M.F., *et al.* (2015). APOBEC family mutational signatures are associated with poor prognosis translocations in multiple myeloma. Nat Commun *6*, 6997.
Weinhold, N., Meissner, T., Johnson, D.C., Seckinger, A., Moreaux, J., Forsti, A., Chen, B., Nickel, J., Chubb, D., Rawstron, A.C., *et al.* (2015). The 7p15.3 (rs4487645) association for multiple myeloma shows strong allele-specific regulation of the MYC-interacting gene CDCA7L in malignant plasma cells. Haematologica *100*, e110-113.
Zhu, Z., Zhang, F., Hu, H., Bakshi, A., Robinson, M.R., Powell, J.E., Montgomery, G.W., Goddard, M.E., Wray, N.R., Visscher, P.M., *et al.* (2016). Integration of summary data from GWAS and eQTL studies predicts complex trait gene targets. Nature genetics *48*, 481-487.
